# In silico discovery of blood cell macromolecular associations

**DOI:** 10.1186/s12863-022-01077-3

**Published:** 2022-07-26

**Authors:** Kaare M. Gautvik, Daniel Sachse, Alexandra C. Hinton, Ole K. Olstad, Douglas P. Kiel, Yi-Hsiang Hsu, Tor P. Utheim, Christine W. Lary, Sjur Reppe

**Affiliations:** 1 Unger-Vetlesen Institute, Lovisenberg Diaconal Hospital, Lovisenberggata 17, 0456 Oslo, Norway; 2 Department of Medical Biochemistry, Oslo University Hospital, Oslo, Norway; 3 Maine Medical Center Research Institute, Scarborough, ME USA; 4 Department of Medicine, The Hinda and Arthur Marcus Institute for Aging Research, Beth Israel Deaconess Medical Center and Harvard Medical School and Broad Institute of MIT & Harvard, Boston, MA USA; 5 Department of Plastic and Reconstructive Surgery, Oslo University Hospital, Oslo, Norway; 6 Department of Ophthalmology, Stavanger University Hospital, Stavanger, Norway; 7 Department of Ophthalmology, Sørlandet Hospital Arendal, Arendal, Norway

**Keywords:** Blood, Transcriptome, Web application, Molecular associations, Microarrays

## Abstract

**Background:**

Physical molecular interactions are the basis of intracellular signalling and gene regulatory networks, and comprehensive, accessible databases are needed for their discovery. Highly correlated transcripts may reflect important functional associations, but identification of such associations from primary data are cumbersome. We have constructed and adapted a user-friendly web application to discover and identify putative macromolecular associations in human peripheral blood based on significant correlations at the transcriptional level.

**Methods:**

The blood transcriptome was characterized by quantification of 17,328 RNA species, including 341 mature microRNAs in 105 clinically well-characterized postmenopausal women. Intercorrelation of detected transcripts signal levels generated a matrix with > 150 million correlations recognizing the human blood RNA interactome. The correlations with calculated adjusted *p*-values were made easily accessible by a novel web application.

**Results:**

We found that significant transcript correlations within the giant matrix reflect experimentally documented interactions involving select ubiquitous blood relevant transcription factors (CREB1, GATA1, and the glucocorticoid receptor (GR, NR3C1)). Their responsive genes recapitulated up to 91% of these as significant correlations, and were replicated in an independent cohort of 1204 individual blood samples from the Framingham Heart Study. Furthermore, experimentally documented mRNAs/miRNA associations were also reproduced in the matrix, and their predicted functional co-expression described. The blood transcript web application is available at http://app.uio.no/med/klinmed/correlation-browser/blood/index.php and works on all commonly used internet browsers.

**Conclusions:**

Using in silico analyses and a novel web application, we found that correlated blood transcripts across 105 postmenopausal women reflected experimentally proven molecular associations. Furthermore, the associations were reproduced in a much larger and more heterogeneous cohort and should therefore be generally representative. The web application lends itself to be a useful hypothesis generating tool for identification of regulatory mechanisms in complex biological data sets.

**Supplementary Information:**

The online version contains supplementary material available at 10.1186/s12863-022-01077-3.

## Background

Regulation of human gene expression relies on functional macromolecules, including transcription factors (TFs), and micro RNAs (miRNAs). TFs may induce or suppress transcription of their target genes, exerted via distinct binding sites and interaction with other signalling molecules [1]. miRNA’s main function is inactivation of mRNAs [2]. Our hypothesis is that highly correlated transcripts in blood and tissue may reflect important functional associations and be a useful tool for hypotheses generation. Signal molecules are often involved in the same pathways and likely to be similarly regulated [3, 4]. We have used the global transcriptome generated from peripheral blood donated by an Oslo cohort of 105 postmenopausal women who were similar with respect to age, ethnicity and health status to generate a large correlation matrix with Pearson r values and modes of significance based on transcription signal values. A web application was developed to explore associations between genes of interest in the dataset.

Coexpressed transcripts tend to reflect co-expressed proteins [5] and we hypothesized that highly correlated transcripts could reflect associations at the protein level. Three ubiquitously expressed TFs known to be functionally important in blood cells were used for testing: cyclic AMP-responsive element (CRE)-binding protein 1 (CREB1), GATA Binding Protein 1 (GATA1) and glucocorticoid receptor (GR). CREB1 is involved in several aspects of hematopoiesis [6–9]. DNA binding and activation of CREB1 depends on its phosphorylation, for example induced by parathyroid hormone (PTH) [10]. Furthermore, expression of *CREB1* in peripheral blood mononuclear cells (PBMC) correlates positively with *CREB1* expression in the postmortem brain of Alzheimer’s patients [11]. Thus, identification of associated transcripts may help to identify novel TFs and their gene targets of functional importance in blood and other tissues. In fact, three datasets of gene expression in immortalized B cells from normal individuals were used to show that correlated transcript levels could be used to predict gene function [12]. GATA1 is essential for erythroid development by regulating the switch of fetal hemoglobin to adult hemoglobin in haemopoietic cells. *GATA1* is a multifunctional gene regulating a plethora of genes, and Encyclopedia of DNA Elements (ENCODE) has registered its response element in nearly 10,000 genes.

The glucocorticoid receptor (GR, *NR3C1*) is also expressed in most tissues and cells, modulating activities of genes involved in cell differentiation/development, metabolism, and immune responses [13]. Natural forms of glucocorticoids or analogs like dexamethasone, all acting through GR, are frequently used medical drugs with direct effects on almost all cell types [14]. Even in physiological concentrations, glucocorticoids regulate major aspects of immune cell functions and are powerful immunosuppressants at pharmacological doses [15].

We also verified the representativeness of our dataset by replicating the top 200 associations in an independent cohort from the Framingham study. Furthermore, we tested if experimentally proven miRNA/mRNA associations, also were statistically correlated in our dataset.

### Construction and content

#### Blood donors

For the postmenopausal blood sampling, Norwegian women (50–86 years, *n* = 105) representing a cohort with varying bone mineral densities (BMDs) and free of primary diseases known to affect the skeleton, were consecutively recruited as described [16]. Blood was collected in the morning from fasting individuals. Postmenopausal women from the Offspring cohort (women aged 40–92, *n* = 1204) participating in The Framingham Study [17, 18] were used as replication cohort. The Framingham Heart Study (FHS) is an ongoing prospective community-based study that includes the children of the original cohort and their spouses, who were enrolled into the Offspring Cohort in 1971. At each FHS examination, age, height, weight and extensive questionnaires were obtained according to standardized protocols. For this analysis, we included Offspring participants who attended examination cycle 8 (2005–2008). Gene expression data was collected for (*n* = 2442) as previously described [18, 19]. These were further filtered on female sex and menopause to achieve the final sample size (*n* = 1204). Of note, hormone replacement therapy was not included in filtration criteria.

#### RNA purification and gene expression analysis

RNA from whole blood was isolated according to the PAXgene Blood RNA Kit manufacturer (BD, Franklin Lakes, NJ, USA), including the optional on-column DNase digestion according to manufacturer’s instructions. RNA from the Oslo and Framingham cohorts were analysed according to manufacturer’s instructions on the Affymetrix Human Gene 1.0 ST GeneChip (Thermo Fisher Scientific, Waltham, MA, USA) which contains ~ 1.4 million probe sets in total. In brief, the Affymetrix Human Exon 1.0 ST Array (Affymetrix, Inc., Santa Clara, CA) was used and gene annotations were obtained from Affymetrix NetAffx Analysis Center (version 31), resulting in ~ 17,000 distinct genes for downstream analysis.

A PCR based method involving LDA cards A and B was used for quantification of ~ 700 microRNAs and other non-coding RNAs in the Oslo cohort according to manufacturer’s instructions (Thermo Fisher Scientific, Waltham, MA, USA).

#### Calculations, statistics and the web application

**Fig. 1:**
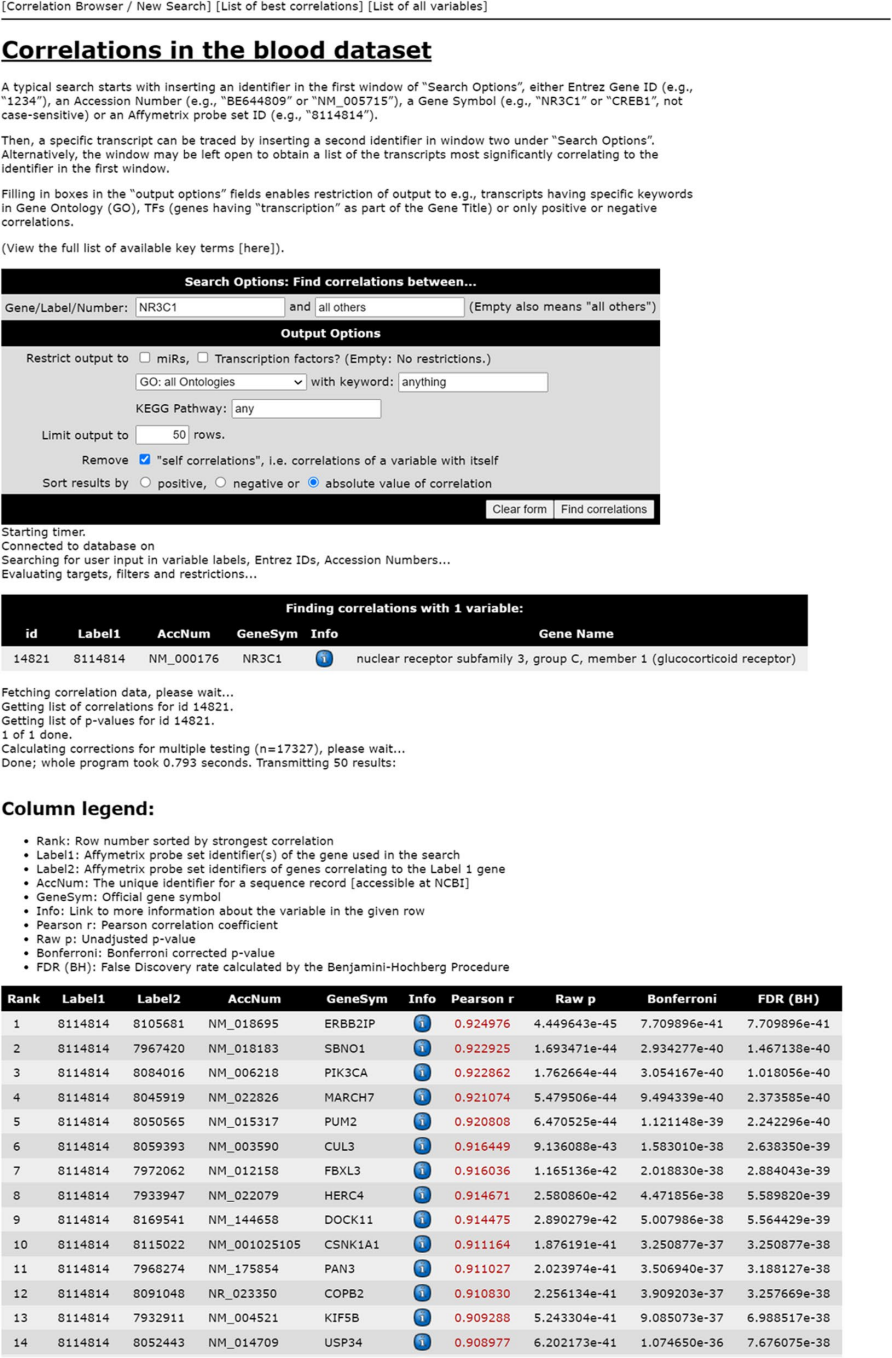
Interface of the web application. A typical search starts with inserting an identifier in the first window of “Search Options”, either Entrez Gene ID (e.g., “1234”), an Accession Number (e.g., “BE644809” or “NM_005715”), a Gene Symbol (e.g., “NR3C1” or “CREB1”, not case-sensitive) or an Affymetrix probe set ID (e.g., “8,114,814”). Then, a specific transcript can be traced by inserting a second identifier in window two under “Search Options”. Alternatively, the window may be left open to obtain a list of the transcripts most significantly correlating to the identifier in the first window. Filling in boxes in the “Output Options” fields enables restriction of output to e.g., transcripts having specific keywords in Gene Ontology (GO), TFs (genes having “transcription” as part of the Gene Title) or only positive or negative correlations

Pearson product-moment correlation coefficients (r) were computed between expressions of all genes (> 17,000 probe sets) across 105 women using log_2_ transformed Affymetrix RMA (Robust Multi-array Average) signal values and inversed PCR Ct values and saved in a database along with their corresponding *p*-values. A web application publicly available at http://app.uio.no/med/klinmed/correlation-browser/blood/index.php was programmed in order to access the database and flexibly search for correlations of interest as previously described [20]. A screenshot of the web application is displayed in Fig. 1. Search results are returned together with raw and Bonferroni-corrected *p*-values and a measure of the false discovery rate (FDR) as estimated by the Benjamini & Hochberg procedure [21]. This procedure has been shown to control FDR when the tests are independent or positively correlated [22]. This assumption is reasonable when identifying differentially expressed genes. The Oslo cohort generated expression data earlier and was the basis for development of the web application. When other expression data became available later, the Framingham data was selected for replication because of similarity with regard to the platform. In the replication analysis, we computed Pearson product moment correlations, followed by Bonferroni correction. Algorithm and methods used in generation of the web application have been more thoroughly described in a previous similar paper [20].

### Utility

#### Verifications

First, we identified the 200 most significantly correlated transcripts for each of the selected transcription factors (CREB1, GATA1, GR) and second, tested if associations identified by the web application reflected experimentally verified interactions. Then, we analysed if the genes harboured the corresponding transcription factor binding sites in their promoters. For this we used ENCODE summarizing results from Chromatin Immunoprecipitation (ChIP) studies by use of the Harmonizome web portal (https://maayanlab.cloud/Harmonizome/gene_set/NR3C1/ENCODE+Transcription+Factor+Targets) [23].

Promoter elements binding CREB1 protein, were identified in 182 of the 200 (91%) topmost CREB1 correlated genes (Table S1), In contrast, when selecting random genes from the same Oslo cohort, 48.3% (SD = 2.1%) were identified as having a CREB1 promoter element (not shown). In all, 13,251 genes have this element as registered by ENCODE, thus, a high fraction of associations are expected for sets of random genes. Similar results were obtained for GATA1 with binding elements found in 73.0% while 34.5% (SD = 6.1) binding elements were found in 200 random genes (Table S2). For GR 35.5% of 200 topmost correlated genes had the GR binding element while random genes had 14.0% (SD = 3.0) binding sites (Table S3). ENCODE have registered 9608 and 4104 genes with binding sites for GATA1 and GR, respectively.

We tested representativeness of the Oslo cohort by checking if the 200 most significant associations in the Oslo cohort were reproduced in blood from postmenopausal women at exam 8 in Framingham Study (*N* = 1204). For CREB1, 180 associations reached significance also in the Framingham cohort (six did not reach significance, and 14 transcripts were undetected) (Table S1). For GATA1 and GR, all, but five and four of the transcripts detected in both cohorts reached statistical significance in the Framingham cohort (Tables S2 and S3, respectively).

#### Evaluation of associations involving microRNAs

To verify whether the web application/matrix can identify also putative miRNA targets, we took advantage of experimentally proven miRNA targets in TarBase 8.0 using DIANA Tools (https://carolina.imis.athena-innovation.gr/diana_tools/web/index.php?r=tarbasev8%2Findex) [24]. For each of the ten miRNAs most highly expressed in peripheral blood based on their PCR Ct-values, the 20 best experimentally verified interacting mRNAs, accessing all cell lines and tissues, were obtained. Out of the ~ 200 interactions/associations, 50 (25%) appeared as nominally significant when analysed in the web application (Table S4). As an alternative evaluation strategy, we selected the top 50 experimentally verified miRNA/mRNA interactions in blood from TarBase 8.0, and found that 30 pairs reached detection level in the Oslo cohort and 13 (43%) of these obtained nominal significance (Table S5).

#### Analysis of the blood interactome employing ingenuity pathway analysis (IPA)

Initially, we tested whether experimentally proven, functional associations mapped by IPA were reproduced in our data. As presented in Table S6, transcripts associating with CREB1 as well as GATA1 were statistically over-represented in functions related to haematological systems within the category “Physiological System Development and Function” As expected, more general functions were attributed to the genes most strongly associated with GR. Furthermore, in the intercorrelation network generated by IPA, all the tested transcriptional regulators (CREB1, GATA1, and GR) had a very central position in the respective top ranked networks (Figs. S1, S2 and S3), supporting that the detected associations were real.

## Discussion

We hypothesized that highly correlated blood cells transcripts could be functionally associated in our dataset and that these associations could be easily assessed by a user-friendly web application. We explored significant macromolecular associations involving CREB1, GATA1 and GR transcription factors. The in silico associations were supported using ENCODE ChIP data from both tissues and cells unrelated to blood, indicating a common functionality irrespective of cell or tissue type. The finding that fewer significant GR correlations (71/200, Table S3) were identified in ENCODE compared to CREB1 (182/200, Table S1) and GATA1 (146/200, Table S2) may be related to GR being able to bind other transcription factors, e.g., those binding to The Activator Protein-1 (AP-1) sites (Fos, Jun and others) without binding directly to DNA [25]. For example, only 62% of dexamethasone induced GR binding sites contained the GR response element when dexamethasone induced transcription was studied in A549 cells [26]. Since thousands of human genes harbour DNA binding elements for the tested transcription factors, we expected to find such elements also in several randomly tested genes used as control, but significantly less. The very high overlap in transcript association between the Framingham and Oslo cohorts, confirmed the validity of the results obtained using in silico analyses. Correlation estimates from the Oslo cohort were generally somewhat higher than in the Framingham dataset, and the difference may be related to a more heterogenous Framingham cohort with respect to age, ethnicity and health status. As expected, experimentally verified mRNA/miRNA associations were not reproduced equally well as mRNA/mRNA associations in our data. This is probably because cell/tissue specific sets of miRNAs are needed to target and degrade mRNAs [2]. Sets of miRNAs targeting specific mRNAs identified in other cohorts and/or tissues may not be present in peripheral blood. Thus, we consider replication of 25% (Table S4) and 43% (Table S5) of verified miRNA/mRNA interactions as satisfactory. The results underscore the usefulness of the in silico approach and web application for detection of miRNA/mRNA associations in peripheral blood, but appear also to have relevance for other tissues. We assume that associations identified are relevant for both sexes, but this needs to be verified.

## Conclusion

The results indicate that in silico analyses using a large correlation matrix containing the blood transcriptome associations in combination with a user-friendly web application, may identify functionally associated macromolecules in blood with relevance also for tissues.

## Supplementary Information

**Additional file 1.**

**Additional file 2.**

## Data Availability

The normalized PCR and Affymetrix signal values used for generation of data accessible in the web application are available in Table S7. The application is available at: http://app.uio.no/med/klinmed/correlation-browser/blood/index.php The primary data used in the web application have also been deposited in NCBI’s Gene Expression Omnibus [27], accessible through GEO Series accession number GSE198941 (https://www.ncbi.nlm.nih.gov/geo/query/acc.cgi?acc=GSE198941).
