# Supplementary material for: In silico discovery of blood cell macromolecular associations
Source: BMC Genom Data. 2022 Jul 26;23:57. doi: 10.1186/s12863-022-01077-3 (PMC9317115; doi:10.1186/s12863-022-01077-3)
Supplement: Supplementary file 1 — Additional file 1. [file 12863_2022_1077_MOESM1_ESM.pdf]

Supplementary

In silico discovery of the blood cell interactome

Kaare M. Gautvik, Daniel Sachse, Alexandra C. Hinton, Ole K. Olstad, Douglas P. Kiel, Yi-Hsiang Hsu, Tor P. Utheim, Christine W. Lary, Sjur Reppe

Table S1. Associations with CREB1

Gene Symbol	Gene Title	NM number	Oslo cohort		Framingham cohort		Comparison	Presence of CREB1 promoter element
			Estimate	P-value[1]	Estimate	P-value[1]		
PKN2	protein kinase N2	NM_006256	0,92	1,3E-38	0,64	1,3E-135	Significant in both cohorts	Yes
SPAG9	sperm associated antigen 9	NM_001130528	0,91	8,4E-36	0,43	6,2E-53	Significant in both cohorts	Yes
JMJD1C	jumonji domain containing 1C	NM_004241	0,9	9,9E-36	NA	NA	Not in Framingham data	Yes
BRWD3	bromodomain and WD repeat domain containing 3	NM_153252	0,9	1,1E-35	0,38	5,3E-40	Significant in both cohorts	Yes
STX7	syntaxin 7	NM_003569	0,9	1,5E-35	0,55	6,4E-92	Significant in both cohorts	Yes
SETX	senataxin	NM_015046	0,9	1,5E-35	0,37	1,3E-38	Significant in both cohorts	Yes
CREBRF	CREB3 regulatory factor	NM_153607	0,9	1,8E-35	0,57	3,5E-101	Significant in both cohorts	Yes
PICALM	phosphatidylinositol binding clathrin assembly protein	NM_007166	0,9	2,1E-35	0,26	2,2E-17	Significant in both cohorts	Yes
BROX	BRO1 domain and CAAX motif containing	NM_144695	0,9	5,0E-35	NA	NA	Not in Framingham data	Yes
SOS2	SOS Ras/Rho guanine nucleotide exchange factor 2	NM_006939	0,9	5,9E-35	0,32	1,5E-27	Significant in both cohorts	Yes
KIAA0232	KIAA0232	NM_014743	0,9	1,2E-34	0,2	6,0E-10	Significant in both cohorts	Yes
AGTPBP1	ATP/GTP binding protein 1	NM_015239	0,9	1,6E-34	0,29	5,2E-23	Significant in both cohorts	Yes
MARCH7	membrane associated ring finger 7	NM_022826	0,9	2,3E-34	0,56	2,0E-98	Significant in both cohorts	Yes
PAN3	PAN3 poly(A) specific ribonuclease subunit	NM_175854	0,9	8,7E-34	0,27	5,3E-20	Significant in both cohorts	Yes
MGEA5	meningioma expressed antigen 5 (hyaluronidase)	NM_012215	0,89	1,3E-33	0,19	7,2E-09	Significant in both cohorts	Yes
VPS8	vacuolar protein sorting 8 homolog (S. cerevisiae)	NM_001009921	0,89	1,3E-33	0,37	3,6E-38	Significant in both cohorts	Yes
MED13	mediator complex subunit 13	NM_005121	0,89	1,5E-33	0,29	1,6E-22	Significant in both cohorts	Yes
MTMR10	myotubularin related protein 10	NM_017762	0,89	3,5E-33	0,28	1,9E-20	Significant in both cohorts	Yes
UBR2	ubiquitin protein ligase E3 component n-recognin 2	NM_015255	0,89	3,7E-33	0,56	3,9E-98	Significant in both cohorts	Yes
MAP3K2	mitogen-activated protein kinase kinase kinase 2	NM_006609	0,89	4,9E-33	0,5	5,2E-76	Significant in both cohorts	No
NIN	ninein (GSK3B interacting protein)	NM_020921	0,89	5,8E-33	0,32	1,5E-28	Significant in both cohorts	Yes
VPS13B	vacuolar protein sorting 13 homolog B (yeast)	NM_017890	0,89	6,1E-33	0,55	4,9E-93	Significant in both cohorts	Yes
IVNS1ABP	influenza virus NS1A binding protein	NM_006469	0,89	8,8E-33	0,4	2,1E-46	Significant in both cohorts	Yes
ATP7A	ATPase, Cu++ transporting, alpha polypeptide	NM_000052	0,89	9,1E-33	0,2	1,9E-10	Significant in both cohorts	Yes
HBP1	HMG-box transcription factor 1	NM_012257	0,89	9,3E-33	0,5	1,3E-74	Significant in both cohorts	Yes
TMEM71	transmembrane protein 71	NM_144649	0,89	1,5E-32	0,29	3,0E-22	Significant in both cohorts	No
SHOC2	SHOC2 leucine-rich repeat scaffold protein	NM_007373	0,89	1,5E-32	0,39	1,5E-43	Significant in both cohorts	Yes
APC	adenomatous polyposis coli	NM_001127511	0,89	1,6E-32	0,49	4,5E-73	Significant in both cohorts	Yes
CYB5R4	cytochrome b5 reductase 4	NM_016230	0,89	1,7E-32	0,45	3,1E-60	Significant in both cohorts	Yes
CFLAR	CASP8 and FADD like apoptosis regulator	NM_003879	0,89	2,2E-32	0,11	4,5E-02	Significant in both cohorts	Yes
RAD21	RAD21 cohesin complex component	NM_006265	0,89	2,4E-32	0,38	3,2E-41	Significant in both cohorts	Yes
USP9X	ubiquitin specific peptidase 9, X-linked	NM_001039590	0,89	3,5E-32	0,33	1,9E-29	Significant in both cohorts	Yes
FNIP1	folliculin interacting protein 1	NM_133372	0,89	3,6E-32	NA	NA	Not in Framingham data	Yes
NUFIP2	nuclear fragile X mental retardation protein interacting	NM_020772	0,89	4,9E-32	0,33	1,2E-29	Significant in both cohorts	Yes
CASP8	caspase 8, apoptosis-related cysteine peptidase	NM_001228	0,89	5,6E-32	0,48	3,7E-67	Significant in both cohorts	Yes
NR3C1	nuclear receptor subfamily 3, group C, member 1	NM_000176	0,89	7,5E-32	0,48	1,5E-68	Significant in both cohorts	Yes
ZFC3H1	zinc finger, C3H1-type containing	NM_144982	0,89	7,9E-32	0,32	4,8E-27	Significant in both cohorts	Yes
RBM39	RNA binding motif protein 39	NM_184234	0,89	7,9E-32	0,27	1,7E-19	Significant in both cohorts	Yes
RICTOR	RPTOR independent companion of MTOR, complex 2	NM_152756	0,89	9,0E-32	0,55	1,0E-93	Significant in both cohorts	Yes
DOCK11	dedicator of cytokinesis 11	NM_144658	0,89	9,4E-32	0,37	7,0E-39	Significant in both cohorts	Yes
KIAA1109	KIAA1109	NM_015312	0,88	1,1E-31	0,31	1,2E-25	Significant in both cohorts	No
ANTXR2	anthrax toxin receptor 2	NM_058172	0,88	1,2E-31	0,54	2,4E-89	Significant in both cohorts	Yes
RRM2B	ribonucleotide reductase M2 B (TP53 inducible)	NM_015713	0,88	1,3E-31	0,35	1,2E-34	Significant in both cohorts	Yes
ITCH	itchy E3 ubiquitin protein ligase	NM_031483	0,88	1,7E-31	0,29	3,2E-22	Significant in both cohorts	Yes
KIDINS220	kinase D-interacting substrate 220kDa	NM_020738	0,88	2,0E-31	NA	NA	Not in Framingham data	Yes
SPOPL	speckle-type POZ protein-like	NM_001001664	0,88	2,0E-31	0,57	1,3E-100	Significant in both cohorts	Yes
KMT2E	lysine (K)-specific methyltransferase 2E	NM_182931	0,88	2,7E-31	0,54	1,6E-91	Significant in both cohorts	Yes
HIPK3	homeodomain interacting protein kinase 3	NM_005734	0,88	3,2E-31	0,37	1,1E-38	Significant in both cohorts	Yes
AKAP10	A kinase (PRKA) anchor protein 10	NM_007202	0,88	4,6E-31	0,23	3,9E-14	Significant in both cohorts	Yes
DDX3X	DEAD (Asp-Glu-Ala-Asp) box helicase 3, X-linked	NM_001356	0,88	4,7E-31	0,26	1,7E-17	Significant in both cohorts	Yes
CEP350	centrosomal protein 350kDa	NM_014810	0,88	4,7E-31	0,47	7,2E-64	Significant in both cohorts	Yes
AHCTF1	AT hook containing transcription factor 1	NM_015446	0,88	4,9E-31	0	1,0E+00	Significant in Oslo only	Yes
TRIM33	tripartite motif containing 33	NM_015906	0,88	5,7E-31	0,57	1,9E-101	Significant in both cohorts	Yes
PTPN12	protein tyrosine phosphatase, non-receptor type 12	NM_002835	0,88	7,8E-31	0,5	1,5E-73	Significant in both cohorts	Yes
LRMP	lymphoid-restricted membrane protein	NM_006152	0,88	8,4E-31	0,4	4,9E-45	Significant in both cohorts	Yes
OSBPL11	oxysterol binding protein-like 11	NM_022776	0,88	9,1E-31	0,56	1,5E-99	Significant in both cohorts	Yes
CUL3	cullin 3	NM_003590	0,88	1,0E-30	0,57	6,3E-102	Significant in both cohorts	Yes
STK4	serine/threonine kinase 4	NM_006282	0,88	1,2E-30	0,32	1,4E-28	Significant in both cohorts	Yes
KBTBD2	kelch repeat and BTB (POZ) domain containing 2	NM_015483	0,88	1,3E-30	0,41	6,1E-48	Significant in both cohorts	Yes
PAPOLA	poly(A) polymerase alpha	NM_032632	0,88	1,3E-30	0,35	3,8E-34	Significant in both cohorts	Yes
UBR5	ubiquitin protein ligase E3 component n-recognin 5	NM_015902	0,88	1,7E-30	0,21	3,9E-11	Significant in both cohorts	Yes
PIK3CA	phosphatidylinositol-4,5-bisphosphate 3-kinase, catalytic	NM_006218	0,88	1,8E-30	0,47	9,0E-65	Significant in both cohorts	Yes
TRAPPC8	trafficking protein particle complex 8	NM_014939	0,88	2,0E-30	0,34	3,4E-32	Significant in both cohorts	Yes
AMD1	adenosylmethionine decarboxylase 1	NM_001634	0,88	2,3E-30	0,52	1,3E-80	Significant in both cohorts	Yes
MKLN1	muskelin 1, intracellular mediator containing kelch motifs	NM_013255	0,88	2,9E-30	0,5	1,6E-73	Significant in both cohorts	Yes
PIK3C3	phosphatidylinositol 3-kinase, catalytic subunit type 3	NM_002647	0,88	3,4E-30	0,31	1,4E-26	Significant in both cohorts	Yes
NIPBL	Nipped-B homolog (Drosophila)	NM_015384	0,88	4,9E-30	0,49	2,6E-71	Significant in both cohorts	Yes
GSK3B	glycogen synthase kinase 3 beta	NM_002093	0,88	5,1E-30	0,38	3,4E-40	Significant in both cohorts	Yes
PHIP	pleckstrin homology domain interacting protein	NM_017934	0,87	7,1E-30	0,46	7,8E-61	Significant in both cohorts	Yes
PUM2	pumilio RNA binding family member 2	NM_015317	0,87	7,8E-30	0,54	5,8E-88	Significant in both cohorts	No
SNX13	sorting nexin 13	NM_015132	0,87	8,9E-30	0,49	6,4E-72	Significant in both cohorts	Yes
ELF2	E74-like factor 2 (ets domain transcription factor)	NM_201999	0,87	1,0E-29	0,4	7,9E-45	Significant in both cohorts	No
BOD1L1	biorientation of chromosomes in cell division 1-like 1	NM_148894	0,87	1,0E-29	0,51	1,9E-77	Significant in both cohorts	Yes
NFAT5	nuclear factor of activated T-cells 5, tonicity-responsive	NM_138714	0,87	1,1E-29	0,33	3,5E-29	Significant in both cohorts	Yes
STXBP5	syntaxin binding protein 5 (tomosyn)	NM_001127715	0,87	1,2E-29	0,42	2,1E-51	Significant in both cohorts	Yes
ARID4B	AT rich interactive domain 4B (RBP1-like)	NM_016374	0,87	1,3E-29	0,48	3,8E-69	Significant in both cohorts	Yes
SRPK2	SRSF protein kinase 2	NM_182691	0,87	1,4E-29	0,51	4,2E-79	Significant in both cohorts	Yes
PHF20L1	PHD finger protein 20-like 1	NM_016018	0,87	1,6E-29	0,53	3,5E-87	Significant in both cohorts	Yes
ITSN2	intersectin 2	NM_006277	0,87	1,6E-29	0,45	1,2E-57	Significant in both cohorts	Yes
STRN	striatin, calmodulin binding protein	NM_003162	0,87	1,8E-29	0,5	2,8E-75	Significant in both cohorts	Yes
PTPRC	protein tyrosine phosphatase, receptor type, C	NM_002838	0,87	1,9E-29	0,5	2,8E-73	Significant in both cohorts	Yes
VPS4B	vacuolar protein sorting 4 homolog B (S. cerevisiae)	NM_004869	0,87	1,9E-29	0,4	3,7E-44	Significant in both cohorts	Yes
ANKRD44	ankyrin repeat domain 44	NM_153697	0,87	1,9E-29	0,37	1,9E-38	Significant in both cohorts	Yes
APAF1	apoptotic peptidase activating factor 1	NM_181861	0,87	2,1E-29	0,32	1,4E-27	Significant in both cohorts	Yes
EXOC1	exocyst complex component 1	NM_001024924	0,87	2,1E-29	0,5	3,1E-75	Significant in both cohorts	Yes
ABI1	abl-interactor 1	NM_005470	0,87	2,4E-29	0,29	3,4E-22	Significant in both cohorts	Yes
USP15	ubiquitin specific peptidase 15	NM_006313	0,87	2,8E-29	0,12	6,0E-03	Significant in both cohorts	Yes
LPGAT1	lysophosphatidylglycerol acyltransferase 1	NM_014873	0,87	2,9E-29	0,42	4,8E-50	Significant in both cohorts	Yes
VPS41	vacuolar protein sorting 41 homolog (S. cerevisiae)	NM_014396	0,87	4,1E-29	0,54	3,8E-90	Significant in both cohorts	Yes
PDS5B	PDS5 cohesin associated factor B	NM_015032	0,87	5,1E-29	0,35	9,1E-33	Significant in both cohorts	Yes
RFWD2	ring finger and WD repeat domain 2, E3 ubiquitin protein	NM_022457	0,87	5,2E-29	0,42	1,2E-50	Significant in both cohorts	Yes
TPR	translocated promoter region, nuclear basket protein	NM_003292	0,87	5,8E-29	0,41	9,1E-49	Significant in both cohorts	Yes
CHD2	chromodomain helicase DNA binding protein 2	NM_001271	0,87	6,4E-29	0,3	5,6E-25	Significant in both cohorts	Yes
OGFRL1	opioid growth factor receptor-like 1	NM_024576	0,87	6,8E-29	0,47	2,0E-63	Significant in both cohorts	Yes
CCPG1	cell cycle progression 1	NM_004748	0,87	6,9E-29	0,29	2,3E-22	Significant in both cohorts	Yes
ADAM10	ADAM metallopeptidase domain 10	NM_001110	0,87	7,8E-29	0,35	6,4E-34	Significant in both cohorts	Yes
DNAJC13	DnaJ (Hsp40) homolog, subfamily C, member 13	NM_015268	0,87	7,9E-29	0,45	2,4E-57	Significant in both cohorts	Yes
PPP1R12A	protein phosphatase 1, regulatory subunit 12A	NM_001143885	0,87	8,4E-29	NA	NA	Not in Framingham data	Yes
TET2	tet methylcytosine dioxygenase 2	NM_017628	0,87	9,5E-29	0,49	1,5E-71	Significant in both cohorts	Yes
RPS6KA5	ribosomal protein S6 kinase, 90kDa, polypeptide 5	NM_004755	0,87	9,6E-29	0,39	8,3E-44	Significant in both cohorts	Yes
ZFYVE16	zinc finger, FYVE domain containing 16	NM_014733	0,87	9,9E-29	NA	NA	Not in Framingham data	No
KIF5B	kinesin family member 5B	NM_004521	0,87	1,0E-28	0,35	2,0E-34	Significant in both cohorts	Yes
LYST	lysosomal trafficking regulator	NM_000081	0,87	1,2E-28	0,54	4,3E-88	Significant in both cohorts	Yes

MAP3K5	mitogen-activated protein kinase kinase kinase 5	NM_005923	0,87	1,2E-28	0,45	5,4E-60	Significant in both cohorts	Yes
STXBP3	syntaxin binding protein 3	NM_007269	0,87	1,3E-28	0,56	8,2E-100	Significant in both cohorts	Yes
PPP3CA	protein phosphatase 3, catalytic subunit, alpha isozyme	NM_000944	0,87	1,3E-28	0,52	1,4E-80	Significant in both cohorts	Yes
AFTPH	aftiphilin	NM_203437	0,87	1,3E-28	0,53	4,7E-87	Significant in both cohorts	Yes
CTBS	chitobiase, di-N-acetyl-	NM_004388	0,87	1,3E-28	0,5	1,1E-73	Significant in both cohorts	Yes
BAZ1A	bromodomain adjacent to zinc finger domain 1A	NM_013448	0,87	1,4E-28	0,33	1,6E-29	Significant in both cohorts	Yes
NT5C2	5-nucleotidase, cytosolic II	NM_012229	0,87	1,5E-28	0,21	3,9E-11	Significant in both cohorts	Yes
CARD8	caspase recruitment domain family, member 8	NM_014959	0,87	1,6E-28	0,07	1,0E+00	Significant in Oslo only	Yes
HERC4	HECT and RLD domain containing E3 ubiquitin protein ligase	NM_022079	0,87	1,7E-28	0,27	4,7E-19	Significant in both cohorts	Yes
GOLGB1	golgin B1	NM_004487	0,87	1,8E-28	0,46	1,2E-61	Significant in both cohorts	Yes
DICER1	dicer 1, ribonuclease type III	NM_177438	0,87	1,8E-28	0,43	4,9E-53	Significant in both cohorts	Yes
RNF169	ring finger protein 169	NM_001098638	0,87	1,8E-28	NA	NA	Not in Framingham data	Yes
LRRFIP1	leucine rich repeat (in FLII) interacting protein 1	NM_001137552	0,87	1,9E-28	NA	NA	Not in Framingham data	Yes
TIPARP	TCDD-inducible poly(ADP-ribose) polymerase	NM_015508	0,87	2,0E-28	0,47	2,2E-65	Significant in both cohorts	Yes
IFNAR1	interferon (alpha, beta and omega) receptor 1	NM_000629	0,86	2,4E-28	0,23	2,4E-13	Significant in both cohorts	Yes
CRLF3	cytokine receptor-like factor 3	NM_015986	0,86	2,9E-28	0,13	5,9E-04	Significant in both cohorts	Yes
NPEPPS	aminopeptidase puromycin sensitive	NM_006310	0,86	3,0E-28	0,22	3,7E-12	Significant in both cohorts	Yes
ST8SIA4	ST8 alpha-N-acetyl-neuraminide alpha-2,8-sialyltransferase 4	NM_005668	0,86	3,4E-28	0,55	2,4E-92	Significant in both cohorts	Yes
KDM5A	lysine (K)-specific demethylase 5A	NM_005056	0,86	3,6E-28	0,1	1,7E-01	Significant in Oslo only	Yes
PCF11	PCF11 cleavage and polyadenylation factor subunit	NM_015885	0,86	3,8E-28	0,31	3,7E-25	Significant in both cohorts	Yes
PIK3CB	phosphatidylinositol-4,5-bisphosphate 3-kinase, catalytic	NM_006219	0,86	3,9E-28	0,42	2,1E-51	Significant in both cohorts	No
RP2	retinitis pigmentosa 2 (X-linked recessive)	NM_006915	0,86	4,1E-28	0,18	2,2E-08	Significant in both cohorts	Yes
RC3H1	ring finger and CCCH-type domains 1	NM_172071	0,86	4,7E-28	NA	NA	Not in Framingham data	No
FBXO38	F-box protein 38	NM_205836	0,86	5,4E-28	NA	NA	Not in Framingham data	Yes
RNASEL	ribonuclease L (2,5-oligoisoadenylate synthetase-dependent)	NM_021133	0,86	6,0E-28	0,29	2,2E-22	Significant in both cohorts	Yes
NEK7	NIMA-related kinase 7	NM_133494	0,86	6,0E-28	0,41	6,0E-48	Significant in both cohorts	Yes
RAB27A	RAB27A, member RAS oncogene family	NM_004580	0,86	6,5E-28	0,07	1,0E+00	Significant in Oslo only	Yes
SCAF11	SR-related CTD-associated factor 11	NM_004719	0,86	8,6E-28	0,34	3,2E-32	Significant in both cohorts	Yes
IFNAR2	interferon (alpha, beta and omega) receptor 2	NM_207585	0,86	8,7E-28	-0,04	1,0E+00	Significant in Oslo only	Yes
CASC4	cancer susceptibility candidate 4	NM_138423	0,86	1,1E-27	0,17	2,6E-07	Significant in both cohorts	Yes
UPF2	UPF2 regulator of nonsense transcripts homolog (yeast)	NM_080599	0,86	1,2E-27	0,32	1,5E-27	Significant in both cohorts	Yes
UBXN4	UBX domain protein 4	NM_014607	0,86	1,3E-27	0,49	3,5E-70	Significant in both cohorts	Yes
IRAK4	interleukin 1 receptor associated kinase 4	NM_001114182	0,86	1,3E-27	0,28	2,7E-21	Significant in both cohorts	Yes
ATF6	activating transcription factor 6	NM_007348	0,86	1,3E-27	0,31	9,0E-27	Significant in both cohorts	Yes
BAZ2B	bromodomain adjacent to zinc finger domain 2B	NM_013450	0,86	1,3E-27	0,49	2,6E-71	Significant in both cohorts	Yes
RAB21	RAB21, member RAS oncogene family	NM_014999	0,86	1,4E-27	0,19	5,3E-09	Significant in both cohorts	Yes
AP3B1	adaptor-related protein complex 3, beta 1 subunit	NM_003664	0,86	1,5E-27	0,57	4,0E-101	Significant in both cohorts	Yes
ZNF217	zinc finger protein 217	NM_006526	0,86	1,8E-27	0,11	1,7E-02	Significant in both cohorts	No
FAM49B	family with sequence similarity 49, member B	BC017297	0,86	1,9E-27	0,23	1,9E-13	Significant in both cohorts	Yes
ELF1	E74-like factor 1 (ets domain transcription factor)	NM_172373	0,86	1,9E-27	0,29	2,9E-23	Significant in both cohorts	No
CD46	CD46 molecule, complement regulatory protein	NM_002389	0,86	1,9E-27	0,46	7,3E-62	Significant in both cohorts	Yes
TLK2	tousled-like kinase 2	NM_006852	0,86	2,1E-27	0,11	1,7E-02	Significant in both cohorts	Yes
PIK3CG	phosphatidylinositol-4,5-bisphosphate 3-kinase, catalytic	NM_002649	0,86	2,2E-27	0,27	8,5E-20	Significant in both cohorts	No
HSDL2	hydroxysteroid dehydrogenase like 2	NM_032303	0,86	2,5E-27	0,33	3,3E-30	Significant in both cohorts	Yes
SP1	Sp1 transcription factor	NM_138473	0,86	2,7E-27	-0,13	6,8E-04	Significant in both cohorts	Yes
TAOK1	TAO kinase 1	NM_020791	0,86	2,9E-27	0,28	1,9E-20	Significant in both cohorts	Yes
MORC3	MORC family CW-type zinc finger 3	NM_015358	0,86	3,5E-27	0,22	2,0E-12	Significant in both cohorts	Yes
RB1	retinoblastoma 1	NM_000321	0,86	3,6E-27	0,27	1,4E-18	Significant in both cohorts	Yes
STK38L	serine/threonine kinase 38 like	NM_015000	0,86	3,9E-27	0,31	2,9E-26	Significant in both cohorts	Yes
NGLY1	N-glycanase 1	NM_018297	0,86	3,9E-27	0,42	7,5E-51	Significant in both cohorts	Yes
TAOK3	TAO kinase 3	NM_016281	0,86	3,9E-27	0,27	1,2E-18	Significant in both cohorts	Yes
OSTF1	osteoclast stimulating factor 1	NM_012383	0,86	4,3E-27	0,09	3,8E-01	Significant in Oslo only	Yes
USP34	ubiquitin specific peptidase 34	NM_014709	0,86	5,2E-27	0,5	1,8E-73	Significant in both cohorts	Yes
ZFX	zinc finger protein, X-linked	NM_003410	0,86	5,6E-27	0,28	1,5E-21	Significant in both cohorts	Yes
CSGALNACT	chondroitin sulfate N-acetylgalactosaminyltransferase 2	NM_018590	0,86	5,7E-27	0,19	9,0E-09	Significant in both cohorts	Yes
PAK2	p21 protein (Cdc42/Rac)-activated kinase 2	NM_002577	0,86	6,4E-27	0,44	5,7E-57	Significant in both cohorts	Yes
RLF	rearranged L-myc fusion	NM_012421	0,86	6,4E-27	0,41	1,4E-46	Significant in both cohorts	Yes
DCP2	decapping mRNA 2	NM_152624	0,85	6,8E-27	0,43	4,5E-54	Significant in both cohorts	Yes
PPTC7	PTC7 protein phosphatase homolog	NM_139283	0,85	7,1E-27	0,15	3,7E-05	Significant in both cohorts	Yes
SLC35F5	solute carrier family 35, member F5	NM_025181	0,85	7,2E-27	0,37	3,0E-38	Significant in both cohorts	Yes
FBXL5	F-box and leucine-rich repeat protein 5	NM_033535	0,85	7,2E-27	0,35	2,0E-34	Significant in both cohorts	Yes
CSNK1A1	casein kinase 1, alpha 1	NM_001025105	0,85	7,2E-27	0,43	1,9E-53	Significant in both cohorts	Yes
CACUL1	CDK2-associated, cullin domain 1	NM_153810	0,85	7,3E-27	0,19	3,1E-09	Significant in both cohorts	Yes
ARIH1	ariadne RBR E3 ubiquitin protein ligase 1	NM_005744	0,85	8,1E-27	0,2	1,8E-10	Significant in both cohorts	Yes
WHSC1L1	Wolf-Hirschhorn syndrome candidate 1-like 1	NM_023034	0,85	8,2E-27	0,37	2,2E-38	Significant in both cohorts	Yes
SYNJ1	synaptojanin 1	NM_003895	0,85	9,0E-27	0,2	1,8E-10	Significant in both cohorts	Yes
KDM7A	lysine (K)-specific demethylase 7A	NM_030647	0,85	9,9E-27	NA	NA	Not in Framingham data	Yes
NUP58	nucleoporin 58kDa	NM_014089	0,85	1,0E-26	0,23	1,2E-13	Significant in both cohorts	No
SEL1L	sel-1 suppressor of lin-12-like (C. elegans)	NM_005065	0,85	1,0E-26	0,26	8,3E-18	Significant in both cohorts	Yes
NRDC	nardilysin convertase	NM_002525	0,85	1,0E-26	0,52	3,9E-83	Significant in both cohorts	No
RPS6KA3	ribosomal protein S6 kinase, 90kDa, polypeptide 3	NM_004586	0,85	1,1E-26	0,2	1,7E-10	Significant in both cohorts	Yes
GDAP2	ganglioside induced differentiation associated protein 2	NM_001135589	0,85	1,1E-26	NA	NA	Not in Framingham data	Yes
VAV3	vav 3 guanine nucleotide exchange factor	NM_006113	0,85	1,3E-26	0,49	3,1E-70	Significant in both cohorts	No
ATG3	autophagy related 3	NM_022488	0,85	1,4E-26	0,45	1,4E-58	Significant in both cohorts	Yes
CLK1	CDC like kinase 1	NR_027856	0,85	1,4E-26	NA	NA	Not in Framingham data	Yes
FBXL3	F-box and leucine-rich repeat protein 3	NM_012158	0,85	1,5E-26	0,33	1,4E-30	Significant in both cohorts	No
SPG11	spastic paraplegia 11 (autosomal recessive)	NM_025137	0,85	1,6E-26	0,24	2,2E-14	Significant in both cohorts	Yes
USP48	ubiquitin specific peptidase 48	NM_032236	0,85	1,9E-26	0,48	4,1E-67	Significant in both cohorts	Yes
PTBP3	polypyrimidine tract binding protein 3	NM_005156	0,85	2,1E-26	0,2	1,2E-09	Significant in both cohorts	Yes
RBM5	RNA binding motif protein 5	NM_005778	0,85	2,2E-26	0,24	1,3E-14	Significant in both cohorts	Yes
CHIC2	cysteine rich hydrophobic domain 2	NM_012110	0,85	2,2E-26	0,26	2,3E-17	Significant in both cohorts	Yes
CLTC	clathrin, heavy chain (Hc)	NM_004859	0,85	2,4E-26	0,32	2,9E-28	Significant in both cohorts	Yes
SNAP23	synaptosome associated protein 23kDa	NM_003825	0,85	2,5E-26	0,13	5,1E-04	Significant in both cohorts	Yes
ERBB2IP	erbb2 interacting protein	NM_018695	0,85	2,6E-26	0,54	3,2E-89	Significant in both cohorts	Yes
TMOD3	tropomodulin 3 (ubiquitous)	NM_014547	0,85	2,7E-26	0,23	3,3E-14	Significant in both cohorts	Yes
CCNL1	cyclin L1	NM_020307	0,85	2,8E-26	0,46	7,0E-61	Significant in both cohorts	Yes
YTHDF3	YTH N(6)-methyladenosine RNA binding protein 3	NM_152758	0,85	2,8E-26	0,49	6,0E-71	Significant in both cohorts	Yes
CHD1	chromodomain helicase DNA binding protein 1	NM_001270	0,85	3,1E-26	0,43	6,4E-53	Significant in both cohorts	Yes
SBNO1	strawberry notch homolog 1 (Drosophila)	NM_018183	0,85	3,1E-26	0,26	2,4E-18	Significant in both cohorts	No
N4BP2L2	NEDD4 binding protein 2-like 2	NM_014887	0,85	3,2E-26	0,21	7,3E-11	Significant in both cohorts	Yes
MCL1	myeloid cell leukemia 1	NM_021960	0,85	3,3E-26	0,31	9,4E-26	Significant in both cohorts	Yes
RBM25	RNA binding motif protein 25	NM_021239	0,85	3,4E-26	0,2	9,2E-10	Significant in both cohorts	Yes
MFSD14A	major facilitator superfamily domain containing 14A	NM_033055	0,85	3,6E-26	0,36	3,4E-35	Significant in both cohorts	No
STAG1	stromal antigen 1	NM_005862	0,85	3,7E-26	0,51	3,8E-77	Significant in both cohorts	Yes
---	---	ENST00000459345	0,85	4,0E-26	NA	NA	Not in Framingham data	NA
MOSPD2	motile sperm domain containing 2	NM_152581	0,85	4,0E-26	0,35	4,8E-34	Significant in both cohorts	Yes
RBPJ	recombination signal binding protein for immunoglobulin	NM_005349	0,85	4,3E-26	0,48	4,6E-68	Significant in both cohorts	Yes

[1]P-values are Bonferroni corrected; NA: not applicable (did not reach detection level in the Framingham cohort); significant correlations are shaded

Table S2. Associations with GATA1

Gene Symbol	Gene Title	NM number	Oslo cohort		Framingham cohort		Comparison	Presence of GATA1 promoter element
			Estimate	P-value[1]	Estimate	P-value[1]		
SPTB	spectrin, beta, erythrocytic	NM 001024858	0,86	5,3E-27	0,61	3,3E-119	Significant in both cohorts	Yes
SHARPIN	SHANK-associated RH domain interactor	NM 030974	0,85	1,9E-26	0,47	2,2E-64	Significant in both cohorts	Yes
ST6GALNAC4	ST6 (alpha-N-acetyl-neuraminyl-2,3-beta-galactosyl-1,3)-N-	NM 175039	0,84	2,9E-25	0,48	2,3E-67	Significant in both cohorts	Yes
DMTN (EPB49)	dematin actin binding protein	NM_001978	0,84	2,9E-24	0,6	3,3E-118	Significant in both cohorts	Yes
RILP	Rab interacting lysosomal protein	NM 031430	0,83	2,7E-23	0,57	3,8E-101	Significant in both cohorts	Yes
SLC6A10PB (SLC6A8)	solute carrier family 6 (neurotransmitter transporter),	NM 005629	0,83	3,6E-23	0,57	1,5E-103	Significant in both cohorts	Yes
ANK1	ankyrin 1, erythrocytic	NM 020476	0,83	3,7E-23	0,59	6,4E-110	Significant in both cohorts	No
SLC25A39	solute carrier family 25, member 39	NM 001143780	0,82	9,2E-23	NA	NA	Not in Framingham data	Yes
CARM1	coactivator-associated arginine methyltransferase 1	NM 199141	0,82	3,1E-22	0,62	9,7E-129	Significant in both cohorts	Yes
FKBP8	FK506 binding protein 8	NM 012181	0,82	4,5E-22	0,5	2,7E-73	Significant in both cohorts	Yes
TNS1	tensin 1	NM 022648	0,81	7,4E-22	0,52	4,3E-83	Significant in both cohorts	No
CDC34	cell division cycle 34	NM 004359	0,81	8,2E-22	0,61	8,3E-120	Significant in both cohorts	Yes
DNAJB2	DnaJ (Hsp40) homolog, subfamily B, member 2	NM 006736	0,81	2,0E-21	0,51	5,3E-80	Significant in both cohorts	Yes
HAGH	hydroxyacylglutathione hydrolase	NM 005326	0,8	2,1E-20	NA	NA	Not in Framingham data	Yes
HBM	hemoglobin, mu	NM 001003938	0,8	4,8E-20	0,47	9,9E-66	Significant in both cohorts	Yes
PRR5	proline rich 5 (renal)	NM 181333	0,79	7,6E-20	NA	NA	Not in Framingham data	No
ATP6V0C	ATPase, H+ transporting, lysosomal 16kDa, V0 subunit c	NM 001694	0,79	1,0E-19	NA	NA	Not in Framingham data	Yes
MAF1	MAF1 homolog, negative regulator of RNA polymerase III	NM 032272	0,79	1,1E-19	0,49	1,5E-70	Significant in both cohorts	Yes
NFIX	nuclear factor I/X (CCAAT-binding transcription factor)	NM 002501	0,79	3,6E-19	0,55	6,2E-92	Significant in both cohorts	Yes
FAM210B (C20orf108)	family with sequence similarity 210, member B	NM 080821	0,79	3,8E-19	0,4	4,6E-45	Significant in both cohorts	Yes
SLC7A5	solute carrier family 7 (amino acid transporter light chain, L	NM 003486	0,78	9,2E-19	0,53	5,4E-86	Significant in both cohorts	Yes
GUCD1 (C22ORF13)	guanylyl cyclase domain containing 1	NM 031444	0,78	2,7E-18	0,63	6,7E-132	Significant in both cohorts	Yes
HDGF	hepatoma-derived growth factor	NM 004494	0,78	3,0E-18	0,55	1,6E-93	Significant in both cohorts	Yes
AP2A1	adaptor-related protein complex 2, alpha 1 subunit	NM 014203	0,78	3,3E-18	0,58	1,1E-104	Significant in both cohorts	Yes
TMEM63B	transmembrane protein 63B	NM 018426	0,78	3,7E-18	NA	NA	Not in Framingham data	Yes
MCOLN1	mucolipin 1	NM 020533	0,77	6,1E-18	0,61	2,2E-120	Significant in both cohorts	No
RBM38	RNA binding motif protein 38	NM 017495	0,77	1,2E-17	0,57	1,2E-102	Significant in both cohorts	Yes
ZER1	zyg-11 related, cell cycle regulator	NM 006336	0,77	1,5E-17	0,64	2,0E-139	Significant in both cohorts	No
UBXN6	UBX domain protein 6	NM_025241	0,77	1,9E-17	0,59	1,6E-112	Significant in both cohorts	No
POLL	polymerase (DNA directed), lambda	NM 013274	0,77	2,0E-17	0,51	8,4E-79	Significant in both cohorts	Yes
ADIPOR1	adiponectin receptor 1	NM 015999	0,77	2,7E-17	NA	NA	Not in Framingham data	Yes
RNF10	ring finger protein 10	NM 014868	0,77	2,8E-17	0,59	3,1E-110	Significant in both cohorts	Yes
IGF2BP2	insulin-like growth factor 2 mRNA binding protein 2	NM 006548	0,76	3,8E-17	0,34	1,6E-31	Significant in both cohorts	No
MXI1	MAX interactor 1, dimerization protein	NM 005962	0,76	5,2E-17	0,38	8,9E-40	Significant in both cohorts	No
MFSD2B	major facilitator superfamily domain containing 2B	NM 001080473	0,76	5,4E-17	NA	NA	Not in Framingham data	Yes
GLRX5	glutaredoxin 5	NM 016417	0,76	6,9E-17	0,5	1,3E-74	Significant in both cohorts	Yes
MARCH2	membrane associated ring finger 2	NM 016496	0,76	8,4E-17	0,04	1,0E+00	Significant in Oslo only	No
RNF123	ring finger protein 123	NM 022064	0,76	1,1E-16	0,55	7,6E-95	Significant in both cohorts	Yes
OSBP2	oxysterol binding protein 2	NM 030758	0,76	1,7E-16	0,51	1,9E-77	Significant in both cohorts	No
KLF1	Kruppel-like factor 1 (erythroid)	NM 006563	0,76	2,1E-16	0,46	3,2E-63	Significant in both cohorts	Yes
E2F2	E2F transcription factor 2	NM 004091	0,75	2,8E-16	0,49	6,3E-70	Significant in both cohorts	Yes
TMOD1	tropomodulin 1	NM 003275	0,75	2,9E-16	0,46	5,1E-63	Significant in both cohorts	Yes
HPS1	Hermansky-Pudlak syndrome 1	NM 000195	0,75	3,0E-16	0,51	2,3E-79	Significant in both cohorts	No
GPR146	G protein-coupled receptor 146	NM 138445	0,75	3,6E-16	0,49	4,0E-72	Significant in both cohorts	Yes
TMEM86B	transmembrane protein 86B	NM 173804	0,75	3,7E-16	0,42	6,7E-50	Significant in both cohorts	Yes
SOX6	SRY box 6	NM 017508	0,75	4,4E-16	0,42	5,0E-50	Significant in both cohorts	Yes
SLC4A1	solute carrier family 4 (anion exchanger), member 1 (Diego	NM 000342	0,75	4,7E-16	0,61	1,8E-122	Significant in both cohorts	Yes
SIAH2	siah E3 ubiquitin protein ligase 2	NM 005067	0,75	4,8E-16	0,46	2,0E-62	Significant in both cohorts	Yes
XK	X-linked Kx blood group	NM 021083	0,75	5,8E-16	0,46	5,9E-61	Significant in both cohorts	Yes
GMPR	guanosine monophosphate reductase	NM 006877	0,75	6,9E-16	0,58	4,4E-105	Significant in both cohorts	Yes
LYL1	lymphoblastic leukemia associated hematopoiesis	NM 005583	0,75	1,0E-15	0,13	1,1E-03	Significant in both cohorts	Yes
ELOF1	ELF1 homolog, elongation factor 1	NM 032377	0,74	1,7E-15	0,41	9,0E-47	Significant in both cohorts	Yes
FAM117A	family with sequence similarity 117, member A	NM 030802	0,74	3,7E-15	0,55	1,1E-95	Significant in both cohorts	Yes
MAP2K3	mitogen-activated protein kinase kinase 3	NM_002756	0,74	4,0E-15	0,54	6,3E-91	Significant in both cohorts	Yes
CDH1	cadherin 1, type 1	NM 004360	0,74	4,5E-15	0,44	2,0E-56	Significant in both cohorts	Yes
R3HDM4 (C19orf22)	R3H domain containing 4	NM 138774	0,74	5,4E-15	0,46	1,5E-60	Significant in both cohorts	Yes
TRIM58	tripartite motif containing 58	NM 015431	0,74	6,5E-15	0,53	7,9E-86	Significant in both cohorts	Yes
SELENBP1	selenium binding protein 1	NM 003944	0,74	6,9E-15	0,6	2,2E-117	Significant in both cohorts	Yes
GUK1	guanylate kinase 1	NM 000858	0,74	7,2E-15	0,52	1,9E-81	Significant in both cohorts	Yes
ROGDI	rogdi homolog	NM 024589	0,73	8,4E-15	0,41	1,3E-46	Significant in both cohorts	Yes
BCL2L1	BCL2-like 1	NM 138578	0,73	8,6E-15	0,55	1,0E-93	Significant in both cohorts	Yes
RANBP10	RAN binding protein 10	NM 020850	0,73	1,0E-14	0,55	8,3E-96	Significant in both cohorts	Yes
TBC1D22B	TBC1 domain family, member 22B	NM 017772	0,73	1,1E-14	0,5	2,8E-73	Significant in both cohorts	Yes
GYPC	glycophorin C (Gerbich blood group)	NM 002101	0,73	1,1E-14	0,53	4,5E-84	Significant in both cohorts	Yes
DPM2	dolichyl-phosphate mannosyltransferase polypeptide 2,	NM 003863	0,73	1,1E-14	0,56	1,1E-99	Significant in both cohorts	Yes
UBE2O	ubiquitin-conjugating enzyme E2O	NM 022066	0,73	1,2E-14	0,51	3,4E-80	Significant in both cohorts	No
CNPPD1 (C2orf24)	cyclin Pas1/PHO80 domain containing 1	NM 015680	0,73	1,2E-14	0,51	1,9E-78	Significant in both cohorts	Yes
FAM214B (KIAA1539)	family with sequence similarity 214, member B	BC004406	0,73	2,1E-14	0,45	5,7E-59	Significant in both cohorts	Yes
RNF187	ring finger protein 187	NM 001010858	0,73	2,1E-14	NA	NA	Not in Framingham data	Yes
FOXO4	forkhead box O4	NM 005938	0,73	2,5E-14	0,47	9,1E-64	Significant in both cohorts	Yes
---	---	ENST00000434251	0,73	3,0E-14	NA	NA	Not in Framingham data	NA
YBX3 (CSDA)	Y box binding protein 3	NM 003651	0,73	3,4E-14	0,41	1,3E-47	Significant in both cohorts	Yes
AHSP	alpha hemoglobin stabilizing protein	NM 016633	0,73	3,5E-14	0,53	1,1E-85	Significant in both cohorts	Yes
CAPN5	calpain 5	NM 004055	0,72	3,9E-14	0,49	4,1E-70	Significant in both cohorts	Yes
RAB11B	RAB11B, member RAS oncogene family	NM 004218	0,72	4,0E-14	0,45	4,1E-59	Significant in both cohorts	No
FURIN	furin (paired basic amino acid cleaving enzyme)	NM 002569	0,72	4,4E-14	0,53	2,4E-86	Significant in both cohorts	Yes
KEL	Kell blood group, metallo-endopeptidase	NM 000420	0,72	4,9E-14	0,44	1,9E-56	Significant in both cohorts	Yes
EPB42	erythrocyte membrane protein band 4.2	NM 000119	0,72	7,2E-14	0,62	9,0E-129	Significant in both cohorts	Yes
MYL4	myosin light chain 4	NM 001002841	0,72	8,0E-14	0,52	5,9E-82	Significant in both cohorts	Yes
TGM2	transglutaminase 2	NM 004613	0,72	9,8E-14	0,55	1,7E-92	Significant in both cohorts	Yes
KLC3	kinesin light chain 3	NM 177417	0,72	1,0E-13	0,52	9,2E-82	Significant in both cohorts	No
---	---	ENST00000444706	0,72	1,1E-13	NA	NA	Not in Framingham data	NA
HMBS	hydroxymethylbilane synthase	NM 000190	0,72	1,2E-13	0,5	9,8E-76	Significant in both cohorts	Yes
TESC	tescalcin	NM 017899	0,72	1,4E-13	0,56	1,5E-97	Significant in both cohorts	No
TRIM10	tripartite motif containing 10	NM 006778	0,71	2,4E-13	0,48	5,3E-69	Significant in both cohorts	No
SLC38A5	solute carrier family 38, member 5	NM 033518	0,71	2,4E-13	0,49	7,5E-73	Significant in both cohorts	Yes
DCAF12	DDB1 and CUL4 associated factor 12	NM 015397	0,71	2,6E-13	0,57	9,2E-103	Significant in both cohorts	Yes
NFIA	nuclear factor I/A	NM 001134673	0,71	5,0E-13	0,37	1,7E-38	Significant in both cohorts	Yes
GRINA	glutamate receptor, ionotropic, N-methyl D-aspartate-	NM 000837	0,71	5,2E-13	0,47	9,7E-66	Significant in both cohorts	Yes
SLC48A1	solute carrier family 48 (heme transporter), member 1	NM 017842	0,71	5,4E-13	0,39	1,1E-42	Significant in both cohorts	Yes
TANGO2 (C22orf25)	transport and golgi organization 2 homolog	NM 152906	0,71	5,8E-13	0,61	5,5E-122	Significant in both cohorts	Yes
---	---	ENST00000410632	0,71	6,2E-13	NA	NA	Not in Framingham data	NA
AK1	adenylate kinase 1	NM 000476	0,71	7,2E-13	0,48	2,8E-67	Significant in both cohorts	Yes
PPP2R5B	protein phosphatase 2, regulatory subunit B, beta	NM 006244	0,7	1,3E-12	0,47	1,1E-64	Significant in both cohorts	Yes
TAL1	T-cell acute lymphocytic leukemia 1	NM 003189	0,7	1,4E-12	0,41	1,7E-47	Significant in both cohorts	Yes
FAM46C	family with sequence similarity 46, member C	NM 017709	0,7	1,5E-12	0,45	1,8E-59	Significant in both cohorts	Yes
KRTAP4-7 (KRTAP4-9)	keratin associated protein 4-7	NM 001146041	0,7	1,7E-12	NA	NA	Not in Framingham data	No
SLC1A5	solute carrier family 1 (neutral amino acid transporter),	NM 005628	0,7	1,7E-12	0,54	3,4E-90	Significant in both cohorts	Yes
TCEB2	transcription elongation factor B (SIII), polypeptide 2	NM 007108	0,7	1,8E-12	0,13	1,7E-03	Significant in both cohorts	Yes
TPGS2 (C18orf10)	tubulin polyglutamylase complex subunit 2	NM 015476	0,7	1,8E-12	0,47	1,0E-63	Significant in both cohorts	No
mar.08	membrane associated ring finger 8	NM 001002265	0,7	2,1E-12	0,46	1,3E-62	Significant in both cohorts	Yes
ALAS2	5-aminolevulinate synthase 2	NM 000032	0,7	2,1E-12	0,52	1,9E-83	Significant in both cohorts	Yes
BLVRB	biliverdin reductase B	NM_000713	0,7	2,3E-12	0,57	9,8E-104	Significant in both cohorts	Yes

PSMF1	proteasome inhibitor subunit 1	NM_178578	0,7	2,4E-12	0,57	3,5E-103	Significant in both cohorts	Yes
HMGN1	high mobility group nucleosome binding domain 1	ENST00000380748	0,7	2,5E-12	NA	NA	Not in Framingham data	Yes
STK11	serine/threonine kinase 11	NM_000455	0,7	3,3E-12	0,48	6,2E-68	Significant in both cohorts	Yes
PTMS	parathymosin	NM_002824	0,69	3,5E-12	0,41	2,2E-48	Significant in both cohorts	Yes
PLEK2	pleckstrin 2	NM_016445	0,69	4,4E-12	0,47	5,6E-65	Significant in both cohorts	Yes
RAD23A	RAD23 homolog A, nucleotide excision repair protein	NM_005053	0,69	6,0E-12	0,56	1,3E-96	Significant in both cohorts	Yes
---	---	ENST00000363109	0,69	6,2E-12	NA	NA	Not in Framingham data	Yes
DNAJC6	DnaJ (Hsp40) homolog, subfamily C, member 6	NM_014787	0,69	7,1E-12	0,38	3,3E-40	Significant in both cohorts	Yes
PHOSPHO1	phosphatase, orphan 1	NM_001143804	0,69	7,5E-12	NA	NA	Not in Framingham data	Yes
ATP1B2	ATPase, Na+/K+ transporting, beta 2 polypeptide	NM_001678	0,69	7,8E-12	0,51	2,2E-79	Significant in both cohorts	Yes
TRAPPC5	trafficking protein particle complex 5	NM_174894	0,69	8,4E-12	0,32	6,0E-28	Significant in both cohorts	Yes
PINK1	PTEN induced putative kinase 1	NM_032409	0,69	1,1E-11	0,57	6,3E-103	Significant in both cohorts	
SNCA	synuclein alpha	NM_000345	0,68	1,4E-11	0,38	1,7E-41	Significant in both cohorts	Yes
RUNDC3A	RUN domain containing 3A	NM_006695	0,68	1,5E-11	0,48	8,2E-67	Significant in both cohorts	No
PNPLA2	patatin-like phospholipase domain containing 2	NM_020376	0,68	1,5E-11	0,44	1,4E-54	Significant in both cohorts	No
SLC6A9	solute carrier family 6 (neurotransmitter transporter,	NM_201649	0,68	1,6E-11	0,43	6,1E-54	Significant in both cohorts	Yes
VWCE	von Willebrand factor C and EGF domains	NM_152718	0,68	2,4E-11	0,47	9,2E-65	Significant in both cohorts	Yes
CCDC124	coiled-coil domain containing 124	NM_138442	0,68	3,4E-11	0,25	8,2E-17	Significant in both cohorts	No
FECH	ferrochelatase	NM_001012515	0,68	3,7E-11	0,47	2,4E-64	Significant in both cohorts	Yes
SEC14L4	SEC14-like lipid binding 4	NM_174977	0,67	5,1E-11	0,37	8,0E-39	Significant in both cohorts	Yes
SPTA1	spectrin, alpha, erythrocytic 1	NM_003126	0,67	5,1E-11	0,39	1,1E-41	Significant in both cohorts	Yes
HBD	hemoglobin, delta	NM_000519	0,67	5,2E-11	0,5	1,8E-73	Significant in both cohorts	Yes
SMIM24 (C19orf77)	small integral membrane protein 24	NM_001136503	0,67	6,4E-11	NA	NA	Not in Framingham data	Yes
NEDD4L	neural precursor cell expressed, developmentally down-	NM_001144967	0,67	1,1E-10	NA	NA	Not in Framingham data	Yes
TSPAN5	tetraspanin 5	NM_005723	0,67	1,2E-10	0,52	1,5E-81	Significant in both cohorts	Yes
PBX1	pre-B-cell leukemia homeobox 1	NM_002585	0,67	1,5E-10	0,42	5,5E-50	Significant in both cohorts	
INPP5K	inositol polyphosphate-5-phosphatase K	NM_130766	0,67	1,7E-10	NA	NA	Not in Framingham data	Yes
SLC25A37	solute carrier family 25 (mitochondrial iron transporter),	NM_016612	0,67	1,8E-10	0,5	6,7E-75	Significant in both cohorts	Yes
---	---	NA	0,66	2,4E-10	NA	NA	Not in Framingham data	NA
SFRP2	secreted frizzled-related protein 2	NM_003013	0,66	2,5E-10	0,3	2,5E-24	Significant in both cohorts	No
PPDPF	pancreatic progenitor cell differentiation and proliferation	NM_024299	0,66	2,5E-10	NA	NA	Not in Framingham data	Yes
ARHGEF12	Rho guanine nucleotide exchange factor (GEF) 12	NM_015313	0,66	2,8E-10	0,41	5,6E-49	Significant in both cohorts	Yes
BABAM1 (C19orf62)	BRISC and BRCA1 A complex member 1	NM_001033549	0,66	3,7E-10	0,53	1,9E-84	Significant in both cohorts	Yes
---	---	NA	0,66	4,3E-10	NA	NA	Not in Framingham data	NA
ASCC2	activating signal cointegrator 1 complex subunit 2	NM_032204	0,66	5,1E-10	0,58	6,2E-107	Significant in both cohorts	Yes
MKRN1	makorin ring finger protein 1	NM_013446	0,65	7,0E-10	0,41	1,3E-48	Significant in both cohorts	Yes
PLVAP	plasmalemma vesicle associated protein	NM_031310	0,65	7,2E-10	0,42	1,6E-51	Significant in both cohorts	Yes
NA	NA	NM_004639	0,65	8,5E-10	0,51	7,6E-77	Significant in both cohorts	Yes
MICAL2	microtubule associated monooxygenase, calponin and LIM	NM_014632	0,65	8,7E-10	0,51	8,0E-77	Significant in both cohorts	No
E2F4	E2F transcription factor 4, p107/p130-binding	NM_001950	0,65	9,6E-10	0,3	1,6E-24	Significant in both cohorts	No
SERF2	small EDRK-rich factor 2	NM_001018108	0,65	1,1E-09	0,28	5,8E-21	Significant in both cohorts	Yes
---	---	ENST00000410622	0,65	1,1E-09	NA	NA	Not in Framingham data	
TSTA3	tissue specific transplantation antigen P35B	NM_003313	0,65	1,4E-09	0,49	5,7E-70	Significant in both cohorts	Yes
HES3	hes family bHLH transcription factor 3	NM_001024598	0,65	1,5E-09	NA	NA	Not in Framingham data	No
YOD1	YOD1 deubiquitinase	NM_018566	0,64	2,2E-09	NA	NA	Not in Framingham data	Yes
PIM1	Pim-1 proto-oncogene, serine/threonine kinase	NM_002648	0,64	2,3E-09	0,48	1,7E-68	Significant in both cohorts	Yes
YPEL3	yippee like 3	NM_031477	0,64	3,0E-09	0,45	5,4E-59	Significant in both cohorts	Yes
SLC14A1	solute carrier family 14 (urea transporter), member 1 (Kidd	NM_001128588	0,64	3,1E-09	0,49	1,6E-70	Significant in both cohorts	Yes
MPP1	membrane protein, palmitoylated 1	NM_002436	0,64	4,0E-09	NA	NA	Not in Framingham data	Yes
SH3GLB2	SH3-domain GRB2-like endophilin B2	NM_020145	0,64	4,5E-09	0,25	6,9E-16	Significant in both cohorts	Yes
PIGQ	phosphatidylinositol glycan anchor biosynthesis class Q	NM_004204	0,64	4,8E-09	0,28	5,8E-21	Significant in both cohorts	Yes
SMOX	spermine oxidase	NM_175839	0,63	6,1E-09	0,47	2,2E-65	Significant in both cohorts	Yes
---	---	ENST00000410524	0,63	6,4E-09	NA	NA	Not in Framingham data	NA
---	---	NA	0,63	6,8E-09	NA	NA	Not in Framingham data	NA
PQLC1	PQ loop repeat containing 1	NM_025078	0,63	7,0E-09	0,22	8,0E-13	Significant in both cohorts	No
MYBL2	v-myb avian myeloblastosis viral oncogene homolog-like 2	NM_002466	0,63	7,1E-09	0,26	1,6E-18	Significant in both cohorts	Yes
---	---	NA	0,63	1,1E-08	NA	NA	Not in Framingham data	
UBL7	ubiquitin-like 7	NM_032907	0,63	1,1E-08	0,43	4,6E-53	Significant in both cohorts	Yes
ERMAP	erythroblast membrane-associated protein (Scianna blood	NM_001017922	0,63	1,1E-08	0,31	1,1E-26	Significant in both cohorts	Yes
---	---	ENST00000410779	0,63	1,2E-08	NA	NA	Not in Framingham data	
MED25	mediator complex subunit 25	NM_030973	0,63	1,2E-08	0,34	1,2E-31	Significant in both cohorts	Yes
YIPF2	Yip1 domain family member 2	NM_024029	0,63	1,4E-08	0,29	1,1E-22	Significant in both cohorts	Yes
BSG	basigin (Ok blood group)	NM_001728	0,63	1,6E-08	0,55	2,8E-93	Significant in both cohorts	Yes
HNRNPU	heterogeneous nuclear ribonucleoprotein U (scaffold	NM_031844	-0,62	1,9E-08	-0,26	1,7E-17	Significant in both cohorts	Yes
SRSF5	serine/arginine-rich splicing factor 5	NM_001039465	-0,62	2,1E-08	-0,13	8,5E-04	Significant in both cohorts	Yes
---	---	ENST00000410468	0,62	2,2E-08	NA	NA	Not in Framingham data	
PDXP	pyridoxal (pyridoxine, vitamin B6) phosphatase	NM_020315	0,62	2,2E-08	0,04	1,0E+00	Significant in Oslo only	No
ANKRD9	ankyrin repeat domain 9	NM_152326	0,62	2,3E-08	0,2	3,8E-10	Significant in both cohorts	Yes
SMIM5 (LOC643008)	small integral membrane protein 5	NM_001162995	0,62	2,3E-08	NA	NA	Not in Framingham data	Yes
SMG1P1	SMG1 pseudogene 1	NM_015092	-0,62	2,6E-08	-0,2	5,1E-10	Significant in both cohorts	No
SRRD	SRR1 domain containing	NM_001013694	0,62	3,0E-08	0,57	9,3E-103	Significant in both cohorts	Yes
---	---	NA	0,62	3,3E-08	NA	NA	Not in Framingham data	NA
H1F0	H1 histone family, member 0	NM_005318	0,62	3,8E-08	0,27	4,1E-19	Significant in both cohorts	Yes
---	---	NA	0,62	4,1E-08	NA	NA	Not in Framingham data	NA
SARAF (TMEM66)	store-operated calcium entry-associated regulatory factor	NM_016127	-0,62	4,2E-08	-0,01	1,0E+00	Significant in Oslo only	Yes
---	---	ENST00000411152	0,62	4,5E-08	NA	NA	Not in Framingham data	NA
---	---	ENST00000411252	0,62	4,8E-08	NA	NA	Not in Framingham data	NA
KANK2	KN motif and ankyrin repeat domains 2	NM_001136191	0,61	5,3E-08	NA	NA	Not in Framingham data	No
CA1	carbonic anhydrase I	NM_001738	0,61	6,3E-08	NA	NA	Not in Framingham data	Yes
NPRL3	NPR3-like, GATOR1 complex subunit	NM_001077350	0,61	6,6E-08	0,45	2,0E-58	Significant in both cohorts	Yes
---	---	NA	0,61	7,2E-08	NA	NA	Not in Framingham data	NA
---	---	NA	0,61	7,3E-08	NA	NA	Not in Framingham data	NA
DNAJA4	DnaJ (Hsp40) homolog, subfamily A, member 4	NM_018602	0,61	7,6E-08	0,46	1,6E-60	Significant in both cohorts	Yes
MRC2	mannose receptor, C type 2	NM_006039	0,61	7,7E-08	0,3	1,1E-23	Significant in both cohorts	No
CRAT	carnitine O-acetyltransferase	NM_000755	0,61	8,1E-08	0,36	9,2E-36	Significant in both cohorts	Yes
MAP2K7	mitogen-activated protein kinase kinase 7	NM_145185	0,61	8,2E-08	0,28	1,6E-20	Significant in both cohorts	Yes
GNAS	GNAS complex locus	NM_000516	0,61	1,0E-07	NA	NA	Not in Framingham data	No
---	---	ENST00000410359	0,61	1,1E-07	NA	NA	Not in Framingham data	NA
ZBED3	zinc finger, BED-type containing 3	NM_032367	0,61	1,2E-07	0,06	1,0E+00	Significant in Oslo only	Yes
GID4 (C17orf39)	GID complex subunit 4 homolog	NM_024052	0,61	1,2E-07	0,41	4,2E-47	Significant in both cohorts	Yes
GSK3A	glycogen synthase kinase 3 alpha	NM_019884	0,61	1,3E-07	0,48	1,2E-67	Significant in both cohorts	Yes
ALDH5A1	aldehyde dehydrogenase 5 family, member A1	NM_170740	0,61	1,4E-07	0,35	8,2E-34	Significant in both cohorts	Yes
HGH1 (C8orf30A)	HGH1 homolog	NM_016458	0,61	1,4E-07	NA	NA	Not in Framingham data	No
SH3GL1	SH3-domain GRB2-like 1	NM_003025	0,6	1,4E-07	0,34	6,9E-31	Significant in both cohorts	No
CYB5R3	cytochrome b5 reductase 3	NM_000398	0,6	2,0E-07	NA	NA	Not in Framingham data	Yes
TM7SF2	transmembrane 7 superfamily member 2	NM_003273	0,6	2,1E-07	0,25	2,2E-16	Significant in both cohorts	No
CISD2	CDGSH iron sulfur domain 2	NM_001008388	0,6	2,3E-07	0,29	3,3E-22	Significant in both cohorts	Yes

[1]P-values are Bonferroni corrected; NA: not applicable (did not reach detection level in the Framingham cohort); significant correlations are shaded

Table S3. Associations with NR3C1/GR

Gene Symbol	Gene Title	NM number	Oslo cohort		Framingham cohort		Comparison	Presence of GR promoter element
			Estimate	P-value[1]	Estimate	P-value[1]		
ERBB2IP	erbb2 interacting protein	NM_018695	0,92	7,7E-41	0,4	4,1E-46	Significant in both cohorts	Yes
SBNO1	strawberry notch homolog 1 (Drosophila)	NM_018183	0,92	2,9E-40	0,29	3,0E-22	Significant in both cohorts	No
PIK3CA	phosphatidylinositol-4,5-bisphosphate 3-kinase, catalytic	NM_006218	0,92	3,1E-40	0,41	6,1E-48	Significant in both cohorts	No
MARCH7	membrane associated ring finger 7	NM_022826	0,92	9,5E-40	0,52	1,9E-80	Significant in both cohorts	Yes
PUM2	pumilio RNA binding family member 2	NM_015317	0,92	1,1E-39	0,49	1,0E-70	Significant in both cohorts	No
CUL3	cullin 3	NM_003590	0,92	1,6E-38	0,46	3,4E-61	Significant in both cohorts	Yes
FBXL3	F-box and leucine-rich repeat protein 3	NM_012158	0,92	2,0E-38	0,23	4,6E-14	Significant in both cohorts	No
HERC4	HECT and RLD domain containing E3 ubiquitin protein ligase 4	NM_022079	0,91	4,5E-38	0,2	6,6E-10	Significant in both cohorts	Yes
DOCK11	dedicator of cytokinesis 11	NM_144658	0,91	5,0E-38	0,32	1,2E-27	Significant in both cohorts	No
CSNK1A1	casein kinase 1, alpha 1	NM_001025105	0,91	3,3E-37	0,4	9,3E-46	Significant in both cohorts	Yes
PAN3	PAN3 poly(A) specific ribonuclease subunit	NM_175854	0,91	3,5E-37	0,23	9,8E-14	Significant in both cohorts	Yes
COPB2	coatomer protein complex subunit beta 2 (beta prime)	NR_023350	0,91	3,9E-37	0,56	3,7E-97	Significant in both cohorts	No
KIF5B	kinesin family member 5B	NM_004521	0,91	9,1E-37	0,28	1,3E-21	Significant in both cohorts	No
USP34	ubiquitin specific peptidase 34	NM_014709	0,91	1,1E-36	0,43	1,3E-53	Significant in both cohorts	Yes
YTHDF3	YTH N(6)-methyladenosine RNA binding protein 3	NM_152758	0,91	1,7E-36	0,36	3,0E-35	Significant in both cohorts	Yes
NIPBL	Nipped-B homolog (Drosophila)	NM_015384	0,91	2,0E-36	0,44	2,1E-55	Significant in both cohorts	Yes
ARIH1	ariadne RBR E3 ubiquitin protein ligase 1	NM_005744	0,91	3,1E-36	0,22	4,4E-12	Significant in both cohorts	Yes
ADAM10	ADAM metallopeptidase domain 10	NM_001110	0,91	3,1E-36	0,34	2,2E-32	Significant in both cohorts	No
MKLN1	muskelin 1, intracellular mediator containing kelch motifs	NM_013255	0,91	3,3E-36	0,44	9,6E-56	Significant in both cohorts	Yes
NEK7	NIMA-related kinase 7	NM_133494	0,91	3,8E-36	0,42	1,9E-51	Significant in both cohorts	No
CEP350	centrosomal protein 350kDa	NM_014810	0,91	4,2E-36	0,47	1,8E-65	Significant in both cohorts	Yes
NUFIP2	nuclear fragile X mental retardation protein interacting	NM_020772	0,91	4,2E-36	0,39	2,8E-42	Significant in both cohorts	Yes
UBR5	ubiquitin protein ligase E3 component n-recognin 5	NM_015902	0,91	6,0E-36	0,3	2,8E-24	Significant in both cohorts	Yes
PCF11	PCF11 cleavage and polyadenylation factor subunit	NM_015885	0,91	8,1E-36	0,2	1,5E-09	Significant in both cohorts	No
PPP3CA	protein phosphatase 3, catalytic subunit, alpha isozyme	NM_000944	0,9	1,1E-35	0,51	1,4E-78	Significant in both cohorts	No
ZFC3H1	zinc finger, C3H1-type containing	NM_144982	0,9	1,4E-35	0,23	4,7E-13	Significant in both cohorts	No
TRAPPC8	trafficking protein particle complex 8	NM_014939	0,9	1,6E-35	0,27	6,7E-20	Significant in both cohorts	Yes
ARFGEF1	ADP-ribosylation factor guanine nucleotide-exchange factor 1	NM_006421	0,9	1,6E-35	0,31	1,2E-25	Significant in both cohorts	No
GDAP2	ganglioside induced differentiation associated protein 2	NM_001135589	0,9	3,4E-35	NA	NA	Not in Framingham data	No
TPR	translocated promoter region, nuclear basket protein	NM_003292	0,9	3,5E-35	0,37	8,6E-39	Significant in both cohorts	No
DDX3X	DEAD (Asp-Glu-Ala-Asp) box helicase 3, X-linked	NM_001356	0,9	3,8E-35	0,22	1,9E-12	Significant in both cohorts	Yes
MED13	mediator complex subunit 13	NM_005121	0,9	4,0E-35	0,28	4,5E-21	Significant in both cohorts	No
ATP7A	ATPase, Cu++ transporting, alpha polypeptide	NM_000052	0,9	4,3E-35	0,13	2,4E-03	Significant in both cohorts	No
CDC73	cell division cycle 73	NM_024529	0,9	6,9E-35	0,43	2,1E-52	Significant in both cohorts	Yes
PDS5B	PDS5 cohesin associated factor B	NM_015032	0,9	8,8E-35	0,23	4,9E-14	Significant in both cohorts	No
MAP3K2	mitogen-activated protein kinase kinase kinase 2	NM_006609	0,9	9,6E-35	0,54	3,7E-91	Significant in both cohorts	No
WAPL (WAPAL)	WAPL cohesin release factor	NM_015045	0,9	9,8E-35	0,27	1,3E-18	Significant in both cohorts	No
VPS8	vacuolar protein sorting 8 homolog (S. cerevisiae)	NM_001009921	0,9	1,1E-34	0,34	2,1E-32	Significant in both cohorts	No
ITCH	itchy E3 ubiquitin protein ligase	NM_031483	0,9	1,1E-34	0,34	1,2E-32	Significant in both cohorts	No
RSBN1L	round spermatid basic protein 1-like	NM_198467	0,9	2,0E-34	0,38	1,0E-40	Significant in both cohorts	No
BCLAF1	BCL2-associated transcription factor 1	NM_014739	0,9	3,4E-34	0,34	5,2E-32	Significant in both cohorts	No
GNAI3	guanine nucleotide binding protein (G protein), alpha	NM_006496	0,9	3,7E-34	0,52	1,4E-80	Significant in both cohorts	No
RBM39	RNA binding motif protein 39	NM_184234	0,9	4,4E-34	0,26	4,0E-17	Significant in both cohorts	Yes
KIDINS220	kinase D-interacting substrate 220kDa	NM_020738	0,9	6,6E-34	NA	NA	Not in Framingham data	No
PHIP	pleckstrin homology domain interacting protein	NM_017934	0,9	6,8E-34	0,39	6,8E-43	Significant in both cohorts	No
IVNS1ABP	influenza virus NS1A binding protein	NM_006469	0,9	6,9E-34	0,38	7,3E-41	Significant in both cohorts	Yes
TNKS2	tankyrase, TRF1-interacting ankyrin-related ADP-ribose	NM_025235	0,9	7,7E-34	0,3	1,7E-24	Significant in both cohorts	No
RAB18	RAB18, member RAS oncogene family	NM_021252	0,9	8,0E-34	0,19	6,8E-09	Significant in both cohorts	No
MON2	MON2 homolog, regulator of endosome-to-Golgi trafficking	NM_015026	0,9	8,3E-34	0,23	5,2E-14	Significant in both cohorts	No
SNX13	sorting nexin 13	NM_015132	0,9	1,0E-33	0,37	9,3E-38	Significant in both cohorts	No
PIK3C3	phosphatidylinositol 3-kinase, catalytic subunit type 3	NM_002647	0,89	1,4E-33	0,3	5,1E-24	Significant in both cohorts	No
KDM5A	lysine (K)-specific demethylase 5A	NM_005056	0,89	1,6E-33	0,13	5,6E-04	Significant in both cohorts	Yes
RAD21	RAD21 cohesin complex component	NM_006265	0,89	1,7E-33	0,35	7,1E-33	Significant in both cohorts	Yes
ZFX	zinc finger protein, X-linked	NM_003410	0,89	2,5E-33	0,25	1,2E-15	Significant in both cohorts	Yes
SYNJ1	synaptojanin 1	NM_003895	0,89	2,7E-33	0,26	2,9E-17	Significant in both cohorts	Yes
SETX	senataxin	NM_015046	0,89	2,8E-33	0,35	2,1E-33	Significant in both cohorts	No
BRWD3	bromodomain and WD repeat domain containing 3	NM_153252	0,89	2,9E-33	0,33	1,8E-30	Significant in both cohorts	No
MED23	mediator complex subunit 23	NM_004830	0,89	3,4E-33	0,41	5,1E-49	Significant in both cohorts	Yes
STAG1	stromal antigen 1	NM_005862	0,89	3,8E-33	0,47	9,5E-64	Significant in both cohorts	No
CRLF3	cytokine receptor-like factor 3	NM_015986	0,89	3,9E-33	0,14	2,5E-04	Significant in both cohorts	No
AMD1	adenosylmethionine decarboxylase 1	NM_001634	0,89	4,8E-33	0,41	1,6E-47	Significant in both cohorts	No
KDM6A	lysine (K)-specific demethylase 6A	NM_021140	0,89	5,6E-33	0,15	2,9E-05	Significant in both cohorts	Yes
RLF	rearranged L-myc fusion	NM_012421	0,89	5,6E-33	0,49	9,9E-72	Significant in both cohorts	Yes
RNF19A	ring finger protein 19A, RBR E3 ubiquitin protein ligase	NM_183419	0,89	5,9E-33	0,25	1,2E-15	Significant in both cohorts	Yes
PKN2	protein kinase N2	NM_006256	0,89	6,8E-33	0,44	3,8E-56	Significant in both cohorts	Yes
SF3B1	splicing factor 3b, subunit 1, 155kDa	NM_012433	0,89	8,0E-33	0,52	2,9E-80	Significant in both cohorts	No
PTPN12	protein tyrosine phosphatase, non-receptor type 12	NM_002835	0,89	1,1E-32	0,44	5,3E-56	Significant in both cohorts	Yes
RAB21	RAB21, member RAS oncogene family	NM_014999	0,89	1,2E-32	0,21	5,8E-11	Significant in both cohorts	No
CLK1	CDC like kinase 1	NR_027856	0,89	1,5E-32	NA	NA	Not in Framingham data	No
RPS6KA3	ribosomal protein S6 kinase, 90kDa, polypeptide 3	NM_004586	0,89	1,6E-32	0,27	1,7E-19	Significant in both cohorts	No
EXOC1	exocyst complex component 1	NM_001024924	0,89	1,6E-32	0,42	2,5E-51	Significant in both cohorts	No
XRCC5	X-ray repair complementing defective repair in Chinese	NM_021141	0,89	2,1E-32	0,63	1,1E-132	Significant in both cohorts	No
STXBP5	syntaxin binding protein 5 (tomosyn)	NM_001127715	0,89	2,4E-32	0,44	2,9E-55	Significant in both cohorts	No
SLC35F5	solute carrier family 35, member F5	NM_025181	0,89	2,6E-32	0,25	1,0E-16	Significant in both cohorts	No
PRKAA1	protein kinase, AMP-activated, alpha 1 catalytic subunit	NM_206907	0,89	2,9E-32	0,48	1,1E-69	Significant in both cohorts	No
ZMYM2	zinc finger, MYM-type 2	NM_003453	0,89	3,0E-32	0,28	7,6E-21	Significant in both cohorts	Yes
AHCTF1	AT hook containing transcription factor 1	NM_015446	0,89	3,3E-32	-0,02	1,0E+00	Significant in Oslo only	No
CLPX	caseinolytic mitochondrial matrix peptidase chaperone	NM_006660	0,89	3,6E-32	0,22	8,4E-12	Significant in both cohorts	No
USP9X	ubiquitin specific peptidase 9, X-linked	NM_001039590	0,89	3,7E-32	0,27	4,2E-19	Significant in both cohorts	Yes
FBXO38	F-box protein 38	NM_205836	0,89	4,0E-32	NA	NA	Not in Framingham data	No
PAPOLA	poly(A) polymerase alpha	NM_032632	0,89	4,1E-32	0,21	5,0E-11	Significant in both cohorts	No
USO1	USO1 vesicle transport factor	NM_003715	0,89	4,8E-32	0,35	3,3E-34	Significant in both cohorts	No
PTPRC	protein tyrosine phosphatase, receptor type, C	NM_002838	0,89	5,0E-32	0,44	1,7E-55	Significant in both cohorts	No
STAG2	stromal antigen 2	NM_001042750	0,89	5,1E-32	0,3	7,0E-25	Significant in both cohorts	Yes
AGTPBP1	ATP/GTP binding protein 1	NM_015239	0,89	5,8E-32	0,24	2,0E-15	Significant in both cohorts	Yes
CREB1	cAMP responsive element binding protein 1	NM_004379	0,89	7,5E-32	0,48	1,5E-68	Significant in both cohorts	Yes
POLR2B	polymerase (RNA) II (DNA directed) polypeptide B, 140kDa	NM_000938	0,89	7,9E-32	0,51	6,5E-78	Significant in both cohorts	No
VPS4B	vacuolar protein sorting 4 homolog B (S. cerevisiae)	NM_004869	0,89	8,6E-32	0,35	2,5E-34	Significant in both cohorts	No
PAFAH1B1	platelet-activating factor acetylhydrolase 1b, regulatory	NM_000430	0,88	9,9E-32	NA	NA	Not in Framingham data	Yes
CLTC	clathrin, heavy chain (Hc)	NM_004859	0,88	1,0E-31	0,37	4,8E-39	Significant in both cohorts	Yes
KBTBD2	kelch repeat and BTB (POZ) domain containing 2	NM_015483	0,88	1,3E-31	0,41	6,2E-49	Significant in both cohorts	Yes
TRIP12	thyroid hormone receptor interactor 12	NM_004238	0,88	1,6E-31	0,45	2,6E-58	Significant in both cohorts	Yes
COL4A3BP	collagen, type IV, alpha 3 (Goodpasture antigen) binding	NM_005713	0,88	1,7E-31	0,62	1,6E-124	Significant in both cohorts	No
TAOK3	TAO kinase 3	NM_016281	0,88	1,8E-31	0,36	2,1E-36	Significant in both cohorts	No
GSK3B	glycogen synthase kinase 3 beta	NM_002093	0,88	2,0E-31	0,39	9,1E-43	Significant in both cohorts	No
IRAK4	interleukin 1 receptor associated kinase 4	NM_001114182	0,88	2,0E-31	0,32	5,7E-27	Significant in both cohorts	No
SPAST	spastin	NM_014946	0,88	2,2E-31	0,4	2,5E-46	Significant in both cohorts	No
ABI1	abl-interactor 1	NM_005470	0,88	2,9E-31	0,19	8,5E-09	Significant in both cohorts	No
SHOC2	SHOC2 leucine-rich repeat scaffold protein	NM_007373	0,88	3,4E-31	0,27	5,7E-19	Significant in both cohorts	No
APPBP2	amyloid beta precursor protein (cytoplasmic tail) binding	NM_006380	0,88	3,4E-31	0,23	7,7E-14	Significant in both cohorts	No

PRPF38B	pre-mRNA processing factor 38B	NM_018061	0,88	4,3E-31	0,37	3,5E-37	Significant in both cohorts	Yes
HECTD1	HECT domain containing E3 ubiquitin protein ligase 1	NM_015382	0,88	6,0E-31	0,26	3,9E-18	Significant in both cohorts	Yes
TMOD3	tropomodulin 3 (ubiquitous)	NM_014547	0,88	8,3E-31	0,25	3,5E-16	Significant in both cohorts	Yes
STK4	serine/threonine kinase 4	NM_006282	0,88	1,1E-30	0,35	1,1E-33	Significant in both cohorts	No
NIN	ninein (GSK3B interacting protein)	NM_020921	0,88	1,2E-30	0,39	9,8E-42	Significant in both cohorts	No
TRIM33	tripartite motif containing 33	NM_015906	0,88	1,3E-30	0,48	3,3E-69	Significant in both cohorts	No
FXR1	fragile X mental retardation, autosomal homolog 1	NM_001013439	0,88	1,4E-30	0,39	6,0E-43	Significant in both cohorts	No
STX7	syntaxin 7	NM_003569	0,88	1,6E-30	0,42	2,1E-50	Significant in both cohorts	No
BROX (C1orf58)	BRO1 domain and CAAX motif containing	NM_144695	0,88	1,6E-30	NA	NA	Not in Framingham data	No
SLC39A9	solute carrier family 39, member 9	NM_018375	0,88	1,7E-30	0,07	1,0E+00	Significant in Oslo only	No
ATP13A3	ATPase type 13A3	NM_024524	0,88	1,9E-30	0,33	1,6E-30	Significant in both cohorts	No
MORC3	MORC family CW-type zinc finger 3	NM_015358	0,88	1,9E-30	0,13	1,3E-03	Significant in both cohorts	Yes
SEL1L	sel-1 suppressor of lin-12-like (C. elegans)	NM_005065	0,88	2,0E-30	0,34	5,9E-31	Significant in both cohorts	No
KIAA1429	KIAA1429	NM_015496	0,88	2,1E-30	0,3	6,0E-25	Significant in both cohorts	Yes
STRN	striatin, calmodulin binding protein	NM_003162	0,88	2,4E-30	0,56	1,3E-98	Significant in both cohorts	Yes
DNAJC13	DnaJ (Hsp40) homolog, subfamily C, member 13	NM_015268	0,88	2,4E-30	0,44	2,4E-56	Significant in both cohorts	No
BZW1 (BZW1L1)	basic leucine zipper and W2 domains 1	NR_026584	0,88	2,5E-30	NA	NA	Not in Framingham data	No
CLK4	CDC like kinase 4	NM_020666	0,88	2,5E-30	0,33	2,2E-29	Significant in both cohorts	No
USP48	ubiquitin specific peptidase 48	NM_032236	0,88	2,9E-30	0,48	8,3E-69	Significant in both cohorts	No
AFTPH	aftiphilin	NM_203437	0,88	3,1E-30	0,52	1,2E-83	Significant in both cohorts	No
AQR	aquarius intron-binding spliceosomal factor	NM_014691	0,88	3,4E-30	0,25	1,0E-15	Significant in both cohorts	No
FAM91A1	family with sequence similarity 91, member A1	NM_144963	0,88	3,5E-30	0,44	8,0E-55	Significant in both cohorts	No
USP15	ubiquitin specific peptidase 15	NM_006313	0,88	3,7E-30	0,21	3,2E-11	Significant in both cohorts	Yes
AZIN1	antizyme inhibitor 1	NM_015878	0,88	4,1E-30	0,09	2,4E-01	Significant in Oslo only	No
CAB39	calcium binding protein 39	NM_016289	0,88	4,3E-30	0,54	1,0E-89	Significant in both cohorts	No
TOPBP1	topoisomerase (DNA) II binding protein 1	NM_007027	0,87	5,6E-30	0,49	1,6E-70	Significant in both cohorts	No
VPS41	vacuolar protein sorting 41 homolog (S. cerevisiae)	NM_014396	0,87	5,8E-30	0,51	1,6E-79	Significant in both cohorts	No
SIRT1	sirtuin 1	NM_012238	0,87	6,1E-30	0,21	2,2E-11	Significant in both cohorts	No
DCP2	decapping mRNA 2	NM_152624	0,87	7,4E-30	0,48	4,1E-67	Significant in both cohorts	No
PIK3CG	phosphatidylinositol-4,5-bisphosphate 3-kinase, catalytic	NM_002649	0,87	7,5E-30	0,42	3,6E-49	Significant in both cohorts	No
ANKRD44	ankyrin repeat domain 44	NM_153697	0,87	7,6E-30	0,46	2,9E-61	Significant in both cohorts	No
STXBP3	syntaxin binding protein 3	NM_007269	0,87	9,2E-30	0,43	7,8E-52	Significant in both cohorts	Yes
PPP1R12A	protein phosphatase 1, regulatory subunit 12A	NM_001143885	0,87	9,7E-30	NA	NA	Not in Framingham data	No
RSF1	remodeling and spacing factor 1	NM_016578	0,87	1,0E-29	0,25	5,4E-16	Significant in both cohorts	Yes
RICTOR	RPTOR independent companion of MTOR, complex 2	NM_152756	0,87	1,1E-29	0,42	2,1E-50	Significant in both cohorts	Yes
ATRX	alpha thalassemia/mental retardation syndrome X-linked	NM_000489	0,87	1,2E-29	0,21	5,2E-11	Significant in both cohorts	No
HOOK3	hook microtubule-tethering protein 3	NM_032410	0,87	1,3E-29	0,42	3,8E-49	Significant in both cohorts	No
RAP1A	RAP1A, member of RAS oncogene family	NM_001010935	0,87	1,4E-29	0,41	8,2E-49	Significant in both cohorts	Yes
NDUFS1	NADH dehydrogenase (ubiquinone) Fe-S protein 1, 75kDa	NM_005006	0,87	1,4E-29	0,39	2,8E-42	Significant in both cohorts	No
TMEM30A	transmembrane protein 30A	NM_018247	0,87	1,5E-29	0,4	4,8E-45	Significant in both cohorts	No
ACAP2	ArfGAP with coiled-coil, ankyrin repeat and PH domains 2	NM_012287	0,87	1,6E-29	0,6	4,9E-115	Significant in both cohorts	No
AGO3 (EIF2C3)	argonaute RISC catalytic component 3	NM_024852	0,87	1,7E-29	0,38	2,3E-39	Significant in both cohorts	No
SOS2	SOS Ras/Rho guanine nucleotide exchange factor 2	NM_006939	0,87	1,8E-29	0,29	2,0E-22	Significant in both cohorts	Yes
KIAA0232	KIAA0232	NM_014743	0,87	1,8E-29	0,11	1,3E-02	Significant in both cohorts	Yes
KIAA1109	KIAA1109	NM_015312	0,87	1,9E-29	0,37	1,1E-37	Significant in both cohorts	No
LTN1	listerin E3 ubiquitin protein ligase 1	NM_015565	0,87	2,0E-29	0,23	4,0E-13	Significant in both cohorts	No
VPS35	VPS35 retromer complex component	NM_018206	0,87	2,0E-29	0,33	1,8E-30	Significant in both cohorts	No
FRYL	FRY like transcription coactivator	NM_015030	0,87	2,1E-29	0,43	2,1E-53	Significant in both cohorts	No
GGNBP2	gametogenetin binding protein 2	NM_024835	0,87	2,2E-29	0,18	6,3E-08	Significant in both cohorts	Yes
SMAD2	SMAD family member 2	NM_005901	0,87	2,2E-29	0,19	4,9E-09	Significant in both cohorts	Yes
CYB5R4	cytochrome b5 reductase 4	NM_016230	0,87	2,3E-29	0,45	7,0E-59	Significant in both cohorts	No
RARS2	arginyl-tRNA synthetase 2, mitochondrial	NM_020320	0,87	2,4E-29	0,4	2,4E-44	Significant in both cohorts	No
CSGALNACT2	chondroitin sulfate N-acetylgalactosaminyltransferase 2	NM_018590	0,87	2,4E-29	0,2	1,6E-10	Significant in both cohorts	No
CCNH	cyclin H	NM_001239	0,87	2,6E-29	0,33	2,1E-30	Significant in both cohorts	Yes
UBXN4	UBX domain protein 4	NM_014607	0,87	2,8E-29	0,47	1,4E-65	Significant in both cohorts	No
MGEA5	meningioma expressed antigen 5 (hyaluronidase)	NM_012215	0,87	2,8E-29	0,24	1,5E-15	Significant in both cohorts	Yes
GOLGB1	golgin B1	NM_004487	0,87	3,0E-29	0,47	3,3E-66	Significant in both cohorts	No
ZNF638	zinc finger protein 638	NM_014497	0,87	3,4E-29	0,37	3,6E-37	Significant in both cohorts	No
BRWD1	bromodomain and WD repeat domain containing 1	NM_033656	0,87	3,7E-29	0,22	6,3E-12	Significant in both cohorts	Yes
FAM214A	family with sequence similarity 214, member A	NM_019600	0,87	3,9E-29	NA	NA	Not in Framingham data	No
FAR1	fatty acyl CoA reductase 1	NM_032228	0,87	4,1E-29	0,25	1,0E-15	Significant in both cohorts	No
UPF2	UPF2 regulator of nonsense transcripts homolog (yeast)	NM_080599	0,87	4,2E-29	0,3	1,7E-24	Significant in both cohorts	Yes
MTM1	myotubularin 1	NM_000252	0,87	4,2E-29	0,26	4,4E-17	Significant in both cohorts	No
sep.07	septin 7	NM_001788	0,87	4,6E-29	0,17	2,4E-07	Significant in both cohorts	Yes
FNIP1	folliculin interacting protein 1	NM_133372	0,87	4,7E-29	NA	NA	Not in Framingham data	No
NAA50	N(alpha)-acetyltransferase 50, NatE catalytic subunit	NM_025146	0,87	4,8E-29	0,39	1,2E-43	Significant in both cohorts	No
UGP2	UDP-glucose pyrophosphorylase 2	NM_001001521	0,87	5,4E-29	0,42	1,7E-50	Significant in both cohorts	Yes
MIR632 (ZNF207)	microRNA 632	NM_001098507	0,87	5,4E-29	0,09	5,0E-01	Significant in Oslo only	No
ATP2C1	ATPase, Ca++ transporting, type 2C, member 1	NM_014382	0,87	5,9E-29	0,34	4,2E-31	Significant in both cohorts	No
UBXN7	UBX domain protein 7	NM_015562	0,87	6,4E-29	0,33	1,8E-29	Significant in both cohorts	No
SLMAP	sarcolemma associated protein	NM_007159	0,87	6,5E-29	0,49	1,6E-70	Significant in both cohorts	Yes
AKAP10	A kinase (PRKA) anchor protein 10	NM_007202	0,87	6,5E-29	0,26	3,4E-17	Significant in both cohorts	Yes
RB1	retinoblastoma 1	NM_000321	0,87	6,6E-29	0,16	1,2E-05	Significant in both cohorts	No
ARID4B	AT rich interactive domain 4B (RBP1-like)	NM_016374	0,87	6,9E-29	0,41	1,5E-46	Significant in both cohorts	No
VPS13B	vacuolar protein sorting 13 homolog B (yeast)	NM_017890	0,87	7,2E-29	0,54	3,9E-89	Significant in both cohorts	Yes
NEMF (SDCCAG1)	nuclear export mediator factor	NM_004713	0,87	7,4E-29	0,17	5,5E-07	Significant in both cohorts	No
PHF3	PHD finger protein 3	NM_015153	0,87	7,8E-29	0,39	1,0E-41	Significant in both cohorts	No
SPAG9	sperm associated antigen 9	NM_001130528	0,87	8,4E-29	0,43	7,9E-52	Significant in both cohorts	Yes
PAK2	p21 protein (Cdc42/Rac)-activated kinase 2	NM_002577	0,87	8,6E-29	0,54	3,2E-89	Significant in both cohorts	No
CHD2	chromodomain helicase DNA binding protein 2	NM_001271	0,87	8,9E-29	0,26	3,6E-17	Significant in both cohorts	No
---	---	ENST00000459345	0,87	9,2E-29	NA	NA	Not in Framingham data	No
OSBPL11	oxysterol binding protein-like 11	NM_022776	0,87	1,0E-28	0,57	2,3E-101	Significant in both cohorts	Yes
HIPK3	homeodomain interacting protein kinase 3	NM_005734	0,87	1,1E-28	0,38	2,8E-40	Significant in both cohorts	Yes
ARID4A	AT rich interactive domain 4A (RBP1-like)	NM_002892	0,87	1,2E-28	0,26	2,5E-18	Significant in both cohorts	No
YME1L1	YME1-like 1 ATPase	NM_139312	0,87	1,3E-28	0,27	7,1E-19	Significant in both cohorts	No
ANKHD1	ankyrin repeat and KH domain containing 1	NM_020690	0,87	1,4E-28	0,56	5,3E-100	Significant in both cohorts	Yes
SENP5	SUMO1/sentrin specific peptidase 5	NM_152699	0,87	1,5E-28	0,01	1,0E+00	Significant in Oslo only	Yes
RAB27A	RAB27A, member RAS oncogene family	NM_004580	0,87	1,5E-28	0,22	1,5E-12	Significant in both cohorts	No
ZNF292	zinc finger protein 292	NM_015021	0,87	1,5E-28	NA	NA	Not in Framingham data	Yes
ITSN2	intersectin 2	NM_006277	0,87	1,5E-28	0,47	1,8E-66	Significant in both cohorts	Yes
RBM25	RNA binding motif protein 25	NM_021239	0,87	1,6E-28	0,17	5,0E-07	Significant in both cohorts	No
IREB2	iron responsive element binding protein 2	NM_004136	0,87	1,7E-28	0,19	5,7E-09	Significant in both cohorts	Yes
CTBS	chitobiase, di-N-acetyl-	NM_004388	0,87	1,8E-28	0,41	1,3E-46	Significant in both cohorts	No
ZFYVE16	zinc finger, FYVE domain containing 16	NM_014733	0,87	1,8E-28	NA	NA	Not in Framingham data	No
ARCN1	archain 1	NM_001655	0,87	1,8E-28	0,19	2,1E-09	Significant in both cohorts	No
UBR3	ubiquitin protein ligase E3 component n-recognin 3 (putative)	NM_172070	0,87	1,9E-28	0,42	5,0E-51	Significant in both cohorts	No
ANGEL2	angel homolog 2 (Drosophila)	NM_144567	0,87	1,9E-28	0,2	2,9E-10	Significant in both cohorts	No
PIK3CB	phosphatidylinositol-4,5-bisphosphate 3-kinase, catalytic	NM_006219	0,87	2,1E-28	0,5	4,6E-74	Significant in both cohorts	No
SCYL3	SCY1-like, kinase-like 3	NM_181093	0,87	2,1E-28	0,28	1,8E-21	Significant in both cohorts	Yes
USP7	ubiquitin specific peptidase 7 (herpes virus-associated)	NM_003470	0,87	2,2E-28	0,17	2,1E-07	Significant in both cohorts	No

[1]P-values are Bonferroni corrected; NA: not applicable (did not reach detection level in the Framingham cohort); significant correlations are shaded

Table S4. Verified associations for top 10 blood expressed miRNAs (verification in all cell lines and tissues)

A	B	C	D	E	F	G	H
hsa-miR-223-3p (hsa-miR-223)				hsa-miR-484			
	TarBase v.8	Correlation browser scores			TarBase v.8	Correlation browser scores	
Gene	pred. score	r	p-value	Gene	pred. score	r	p-value
SCARB1	0,90	-0,22	2,58E-02	AVL9	1,00	0,24	1,40E-02
RASGRP1	0,90	-0,01	8,97E-01	PCDH19	1,00	NA	
DDIT4	0,82	0,04	6,56E-01	SLC20A2	1,00	-0,02	8,33E-01
GFPT1	0,74	0,14	1,47E-01	SNRNP200	0,99	0,25	1,03E-02
RGS1	0,73	NA		DCBLD2	0,99	NA	
MECP2	0,70	-0,21	3,59E-02	SNN	0,99	0,09	3,76E-01
MT1E	0,68	-0,01	9,52E-01	ANAPC7	0,98	0,18	7,33E-02
TTBK2	0,67	-0,24	1,24E-02	KCNJ2	0,98	0,13	1,98E-01
WDR7	0,65	0,30	2,09E-03	C10orf2	0,97	-0,06	5,35E-01
FADS1	0,59	-0,01	9,20E-01	ACVR1B	0,96	0,06	5,52E-01
GDI1	0,57	-0,12	2,09E-01	IFNAR1	0,96	0,21	3,50E-02
CHUK	0,57	0,34	3,99E-04	FAM212B	0,96	NA	
SOX5	0,56	NA		KMT2A	0,96	NA	
SERINC1	0,55	0,35	2,45E-04	NOL9	0,95	0,28	4,40E-03
TMEM69	0,51	0,13	1,76E-01	HIPK1	0,95	0,27	5,82E-03
CPEB4	0,49	0,20	4,62E-02	HOXA5	0,95	NA	
RNF213	0,49	-0,10	2,96E-01	SCARB1	0,90	-0,18	6,62E-02
COX7A2L	0,45	-0,01	9,17E-01	TCHP	0,90	-0,06	5,71E-01
				RASGRP1	0,90	0,23	2,00E-02
				RTN3	0,90	0,02	8,42E-01

I	J	K	L	M	N	O	P
hsa-miR-191-5p (hsa-miR-191)				hsa-miR-19b-3p (hsa-miR-19b)			
	TarBase v.8	Correlation browser scores			TarBase v.8	Correlation browser scores	
Gene	pred. score	r	p-value	Gene	pred. score	r	p-value
FUBP3	0,90	NA		PATL1	1,00	-0,04	7,14E-01
AMMECR1L	0,89	0,05	5,94E-01	SKIDA1	1,00	na	
TMOD2	0,88	-0,05	6,05E-01	RNF111	1,00	0,04	6,87E-01
TJP1	0,84	na		ELOVL5	1,00	0,09	3,42E-01
ZBTB34	0,82	-0,10	2,97E-01	SMARCA2	1,00	-0,03	7,32E-01
TLX3	0,82	0,05	6,14E-01	ATXN1	1,00	-0,01	9,01E-01
DTD2	0,81	na		PCDH10	1,00	na	
CRKL	0,78	0,02	8,69E-01	SLC9A6	1,00	0,17	9,06E-02
CDK6	0,77	0,16	9,73E-02	HBP1	1,00	0,38	7,13E-05
CEBPB	0,75	-0,03	7,42E-01	ZBTB10	1,00	0,07	5,10E-01
WWP1	0,73	0,05	6,16E-01	SIN3B	1,00	-0,38	5,65E-05
WIZ	0,73	0,01	9,17E-01	FBXO32	1,00	-0,07	4,91E-01
EIF3B	0,72	0,04	6,65E-01	PRICKLE2	1,00	na	
HLTF	0,71	0,12	2,27E-01	ADSS	1,00	0,24	1,57E-02
DCAF15	0,69	0,15	1,35E-01	ZBTB18	1,00	na	
PWWP2A	0,68	-0,12	2,24E-01	RAPGEF2	1,00	0,14	1,58E-01
B4GALT6	0,66	na		PNRC1	1,00	0,21	3,07E-02
EGR1	0,66	-0,02	8,53E-01	MYLIP	1,00	0,17	8,09E-02
SIN3B	0,64	0,15	1,23E-01	RORA	1,00	-0,01	9,05E-01
BLOC1S5-TXNDC5	0,64	na		TUB	1,00	na	

Q	R	S	T	U	V	W	X
hsa-miR-150-5p (hsa-miR-150)				hsa-miR-16-5p (hsa-miR-16)			
	TarBase v.8	Correlation browser scores			TarBase v.8	Correlation browser scores	
Gene	pred. score	r	p-value	Gene	pred. score	r	p-value
MTCH2	0,99	0,05	5,95E-01	PRDM4	1,00	0,00	9,99E-01
LDLR	0,98	0,06	5,37E-01	FBXW7	1,00	-0,06	5,70E-01
PDE7A	0,98	0,25	1,14E-02	ASH1L	1,00	-0,21	3,22E-02
MDM4	0,93	-0,02	8,55E-01	TSPYL2	1,00	0,00	9,61E-01
PERP	0,93	0,06	5,14E-01	PLAG1	1,00	na	
SMC3	0,89	-0,02	8,58E-01	FGF2	1,00	0,16	1,03E-01
SAR1A	0,88	0,00	9,66E-01	PAPPA	1,00	na	
AZIN1	0,88	-0,09	3,71E-01	ZBTB34	1,00	-0,04	7,03E-01
CHD2	0,85	-0,09	3,64E-01	USP25	1,00	-0,20	4,01E-02
SP1	0,83	-0,13	1,86E-01	CTTNBP2	1,00	na	
VTI1A	0,80	-0,05	6,45E-01	CACUL1	1,00	na	
FAM46C	0,76	-0,03	7,85E-01	WIPI2	1,00	0,06	5,59E-01
NBEAL1	0,75	0,05	5,89E-01	SLIT2	1,00	na	
MESDC2	0,73	0,15	1,15E-01	SREK1	1,00	na	
CDKN1B	0,72	-0,11	2,53E-01	LSM11	1,00	na	
ATP9A	0,71	-0,12	2,07E-01	CDC37L1	1,00	0,14	1,69E-01
LSM8	0,70	na		GOLGA1	1,00	0,00	9,90E-01
ALDH6A1	0,69	0,20	4,39E-02	WNT3A	1,00	0,11	2,57E-01
ZNF395	0,67	0,25	1,17E-02	SLC20A2	1,00	-0,16	1,01E-01
ZNF625	0,66	0,10	3,20E-01	CD2AP	1,00	-0,03	7,69E-01

Y	Z	AA	AB	AC	AD	AE	AF
hsa-miR-92a-3p (hsa-miR-92a)				hsa-miR-30c-5p (hsa-miR-30c)			
	TarBase v.8	Correlation browser scores			TarBase v.8	Correlation browser scores	
Gene	pred. score	r	p-value	Gene	pred. score	r	p-value
UBE2W	1,00	-0,10	3,22E-01	SNAI1	1,00	na	
MIA3	1,00	0,18	7,37E-02	ERLIN1	1,00	0,15	1,31E-01
ERGIC2	1,00	-0,08	4,42E-01	PFN2	1,00	na	
FBXW7	1,00	-0,01	9,01E-01	PLAGL2	1,00	-0,04	7,13E-01
FNIP2	1,00	0,19	5,17E-02	PDE7A	1,00	0,43	4,95E-06
CD69	1,00	-0,13	1,74E-01	SYNGR3	1,00	-0,21	3,54E-02
KLHDC10	1,00	-0,05	6,30E-01	R3HDM1	1,00	0,28	3,52E-03
PTAR1	1,00	0,10	2,96E-01	TLE1	1,00	-0,12	2,36E-01
MAN2A1	1,00	0,04	6,55E-01	TBC1D10B	1,00	-0,13	1,96E-01
PPP1R37	1,00	na		GLCCI1	1,00	0,20	4,60E-02
DKK3	1,00	-0,05	5,93E-01	MTDH	1,00	0,27	5,99E-03
ARRDC3	1,00	-0,01	9,01E-01	SETD7	1,00	0,12	2,16E-01
MAP2K4	1,00	-0,05	6,38E-01	PPARGC1B	1,00	-0,34	3,71E-04
ITGA5	1,00	0,36	2,00E-04	CCNE2	1,00	na	
KLF4	1,00	0,16	9,56E-02	GALNT7	1,00	0,36	1,88E-04
TEF	1,00	-0,05	5,96E-01	MEX3B	1,00	-0,25	8,78E-03
GRAMD1B	1,00	0,12	2,31E-01	DCUN1D3	1,00	0,01	8,81E-01
FMN2	1,00	na		PIP4K2A	1,00	-0,20	4,18E-02
LHFPL2	1,00	0,03	7,35E-01	BRWD3	1,00	0,33	6,79E-04
JMY	1,00	0,16	1,10E-01	LCLAT1	1,00	0,28	3,61E-03

AG	AH	AI	AJ	AK	AL	AM	AN
hsa-miR-30b-5p (hsa-miR-30b)				hsa-miR-486-5p			
	TarBase v.8	Correlation browser scores			TarBase v.8	Correlation browser scores	
Gene	pred. score	r	p-value	Gene	pred. score	r	p-value
ERLIN1	1,00	0,15	1,34E-01	SRSF3	1,00	-0,23	1,91E-02
PFN2	1,00	na		BTAF1	0,99	-0,04	6,92E-01
PDE7A	1,00	0,39	3,08E-05	TANC1	0,99	na	
PLAGL2	1,00	-0,03	7,94E-01	PLAGL2	0,99	0,21	3,16E-02
PIP4K2A	1,00	-0,24	1,42E-02	PTEN	0,99	-0,27	5,04E-03
R3HDM1	1,00	0,18	6,18E-02	ST6GALNAC6	0,99	0,19	5,80E-02
SYNGR3	1,00	-0,17	7,47E-02	CREBL2	0,96	-0,17	8,82E-02
TLE1	1,00	-0,17	9,22E-02	ZNF367	0,93	0,11	2,58E-01
GLCCI1	1,00	0,11	2,70E-01	RBM12	0,93	na	
MTDH	1,00	0,23	1,90E-02	EMP1	0,91	na	
PPARGC1B	1,00	-0,31	1,16E-03	PYCR2	0,86	0,14	1,48E-01
GALNT7	1,00	0,39	3,06E-05	NR2F1	0,83	-0,06	5,65E-01
DCUN1D3	1,00	0,07	4,81E-01	ITGB8	0,82	na	
CCNE2	1,00	na		TFAM	0,81	-0,29	2,98E-03
BRWD3	1,00	0,36	1,94E-04	G3BP2	0,81	-0,10	3,28E-01
MEX3B	1,00	-0,22	2,31E-02	GXYLT1	0,80	-0,22	2,62E-02
KLHL20	1,00	0,24	1,35E-02	RBM12B	0,78	-0,21	3,44E-02
DESI2	1,00	na		G3BP1	0,78	-0,01	0,92
ZNRF1	1,00	-0,21	2,90E-02	C7orf13	0,77	-0,06	5,30E-01
MAML1	1,00	-0,35	2,15E-04	PITPNA	0,75	-0,08	4,39E-01

The ten toppmost expressed peripheral blood miRNAs was identified based on PCR ct-values. Then regulation of/by other genes was identified from TarBase 8.0 using DIANA Tools as described below, thereby identifying experimentally verified interactions (considering all cell lines and tissues) as prediction score (column k, p, u.....).
The 20 miRNAs/gene pairs with topmost TarBase prediction scores (column B. F, J.....) were then compared as indicated with Pearson correlation r and p values (columns C+D, G+H, K+L,.....) from the peripheral blood correlation browser.

Assesment of gene regulation using TarBase
TarBase v.8 prediction score was assessed using the function TarBase v.8 in DIANA tools (http://carolina.imis.athena-innovation.gr/diana_tools/web/index.php?r=tarbasev8%2Findex)
with the following setting:
Species: Homo sapiens
(All other settings remained un-ticked)

Table S5. Topmost verified peripheral blood mRNA/miRNA interactions versus associations in the Oslo cohort

Output from TarBase v.8							Corresponding output from the Correlation browser	
Gene name	miRNA	Experiments throughput	Publications	Cell lines	Tissues	Pred. Score	r	p-value
ITCH	hsa-miR-146a-5p	low: 0 high: 1	1	1	1	0,94	-0,31	1,54E-03
PTAR1	hsa-miR-146a-5p	low: 0 high: 2	1	1	1	0,89	-0,22	2,54E-02
RBL1	hsa-miR-146a-5p	low: 0 high: 1	1	1	1	0,85	-0,17	8,00E-02
BACH1	hsa-miR-20a-3p	low: 1 high: 0	1	1	1	0,83	0,09	3,59E-01
BACH1	hsa-miR-17-3p	low: 1 high: 0	1	1	1	0,80	0,08	4,39E-01
PELI1	hsa-miR-106b-5p	low: 1 high: 0	1	1	1	0,79	0,15	1,38E-01
PELI1	hsa-miR-20a-5p	low: 1 high: 0	1	1	1	0,78	0,07	4,93E-01
SRSF1	hsa-miR-146a-5p	low: 0 high: 1	1	1	1	0,76	-0,26	7,53E-03
CLIC4	hsa-miR-25-3p	low: 1 high: 0	1	1	1	0,73	-0,04	6,72E-01
LY75	hsa-miR-146a-5p	low: 1 high: 0	1	1	1	0,72	-0,17	7,50E-02
TNIK	hsa-miR-146a-5p	low: 0 high: 1	1	1	1	0,72	-0,02	8,11E-01
SESTD1	hsa-miR-146a-5p	low: 0 high: 1	1	1	1	0,72	-0,24	1,23E-02
DAZAP2	hsa-miR-17-5p	low: 1 high: 0	1	1	1	0,70	-0,05	6,04E-01
DAZAP2	hsa-miR-93-5p	low: 1 high: 0	1	1	1	0,69	0,05	6,27E-01
LY75	hsa-miR-20a-5p	low: 1 high: 0	1	1	1	0,67	-0,07	4,63E-01
LY75	hsa-miR-106b-5p	low: 1 high: 0	1	1	1	0,67	0,09	3,73E-01
BACH1	hsa-miR-25-3p	low: 1 high: 0	1	1	1	0,66	0,08	3,94E-01
WDR36	hsa-miR-146a-5p	low: 0 high: 1	1	1	1	0,65	-0,22	2,41E-02
BACH1	hsa-miR-146a-5p	low: 1 high: 0	1	1	1	0,64	-0,36	1,59E-04
SPEN	hsa-miR-146a-5p	low: 0 high: 1	1	1	1	0,63	-0,30	1,92E-03
PELI1	hsa-miR-25-3p	low: 1 high: 0	1	1	1	0,62	0,13	1,97E-01
GIMAP4	hsa-miR-146a-5p	low: 0 high: 1	1	1	1	0,62	-0,25	1,05E-02
PDS5A	hsa-miR-146a-5p	low: 0 high: 1	1	1	1	0,60	-0,22	2,30E-02
MED13	hsa-miR-146a-5p	low: 0 high: 1	1	1	1	0,60	-0,26	6,72E-03
SUPT16H	hsa-miR-146a-5p	low: 0 high: 1	1	1	1	0,60	-0,27	4,79E-03
DAZAP2	hsa-miR-20a-5p	low: 1 high: 0	1	1	1	0,59	0,00	9,68E-01
KBTBD6	hsa-miR-146a-5p	low: 0 high: 1	1	1	1	0,59	0,14	1,55E-01
DAZAP2	hsa-miR-106b-5p	low: 1 high: 0	1	1	1	0,59	0,04	7,09E-01
LNPEP	hsa-miR-146a-5p	low: 0 high: 1	1	1	1	0,59	-0,24	1,50E-02
HOXB8	hsa-miR-146a-5p	low: 0 high: 1	1	1	1	0,59	0,22	2,59E-02

The topmost peripheral blood experimentally verified miRNA/mRNA interactions with highest predictive score were identified using the function TarBase v.8 in DIANA tools (leftmost panel). The corresponding miRNAs/gene pairs with Pearson r and p values, calculated in the correlation browser, are shown in the rightmost panel. Nominally significant associations ($p < 0.05$) are shaded.

Table S6. Results from Ingenuity Pathway Analysis within the category "Physiological System Development and Function" for the transcripts associated with transcription factors CREB1, GATA1 and GR

Name	p-value range	# Molecules
**CREB1**		
Embryonic Development	4.30E-03 - 9.11E-06	18
Hematological System Development and Function	4.47E-03 - 9.11E-06	17
Hematopoiesis	4.30E-03 - 9.11E-06	13
Lymphoid Tissue Structure and Development	4.30E-03 - 9.11E-06	17
Organ Development	4.30E-03 - 9.11E-06	17
**GATA1**		
Hematological System Development and Function	3.30E-02 - 1.18E-09	15
Hematopoiesis	3.30E-02 - 1.18E-09	10
Tissue Morphology	3.42E-02 - 1.18E-09	20
Connective Tissue Development and Function	3.52E-02 - 1.22E-04	16
Organismal Development	3.52E-02 - 1.22E-04	22
**GR**		
Organismal Survival	2.24E-03 - 1.29E-06	23
Embryonic Development	4.47E-03 - 3.31E-05	12
Organismal Development	4.47E-03 - 3.31E-05	18
Tissue Morphology	4.47E-03 - 3.31E-05	13
Nervous System Development and Function	4.47E-03 - 1.36E-04	5

The indicated transcription factors and respective 50 topmost associated genes (Table 1, 2 or 3) were subjected to «core analysis» including direct and indirect relationships and default settings/filters.

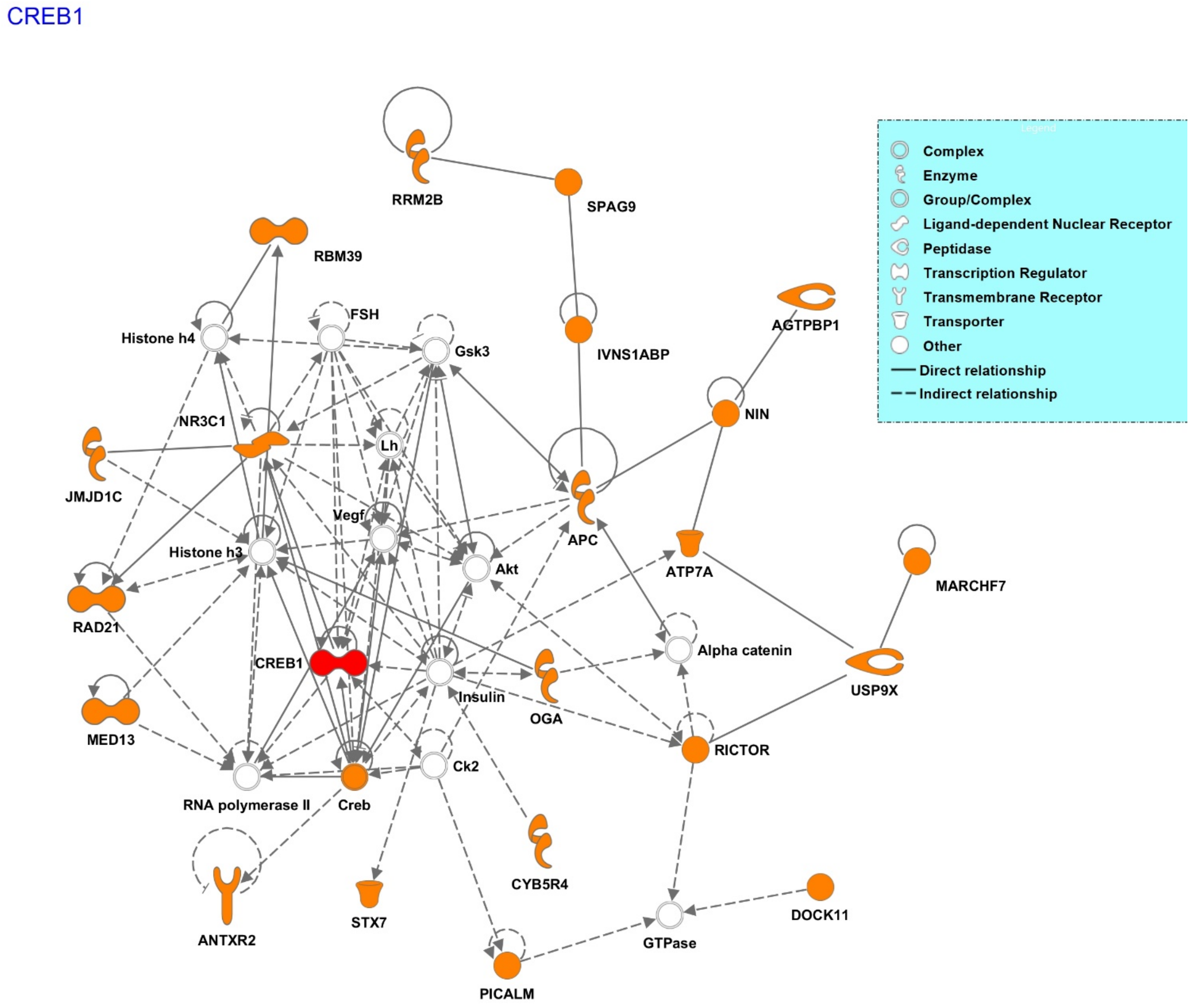

© 2000-2021 QIAGEN. All rights reserved.

Figure S1. Top network calculated by IPA when CREB1 and its 50 topmost associated genes were subjected to «core analysis» including direct and indirect relationships and default settings/filters. Orange symbols indicate genes among the 50 topmost CREB1 associated.

GATA1

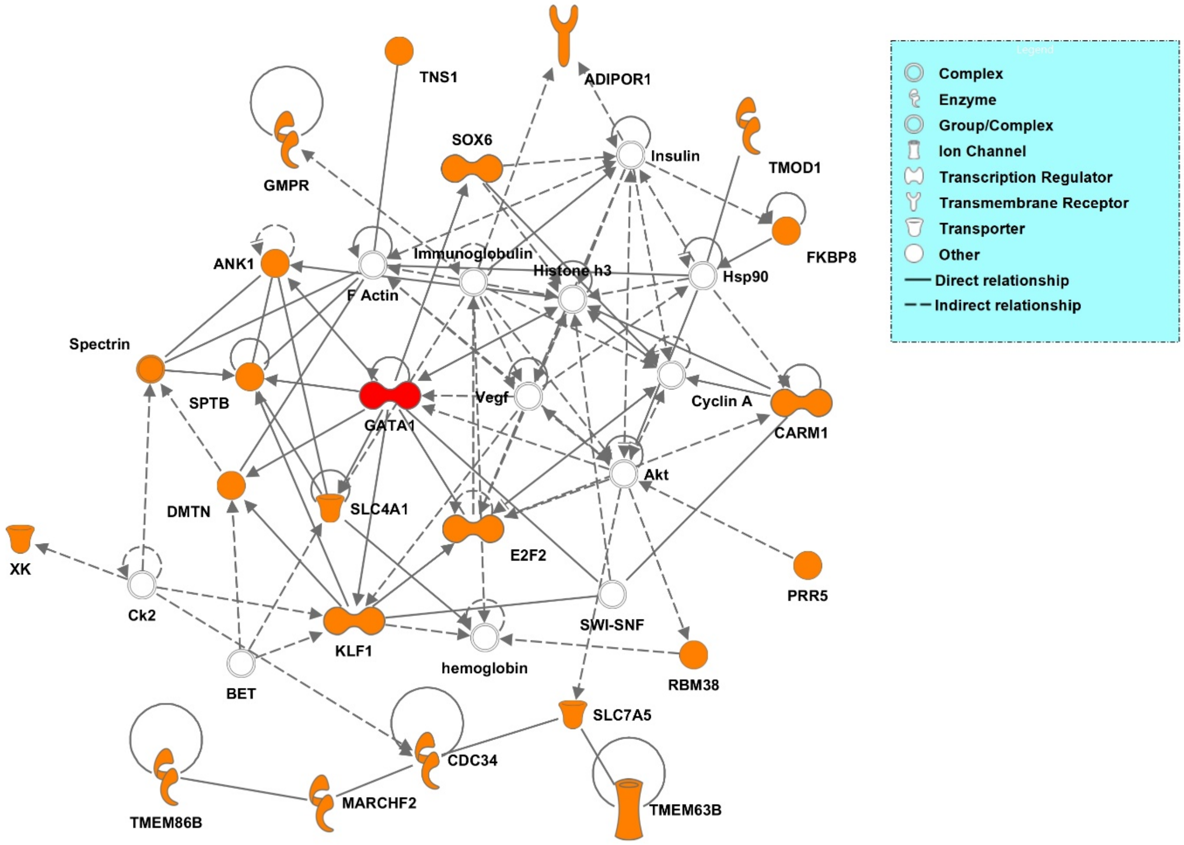

© 2000-2021 QIAGEN. All rights reserved.

Figure S2. Top network calculated by IPA when GATA1 and its 50 topmost associated genes were subjected to «core analysis» including direct and indirect relationships and default settings/filters. Orange symbols indicate genes among the 50 topmost GATA1 associated.

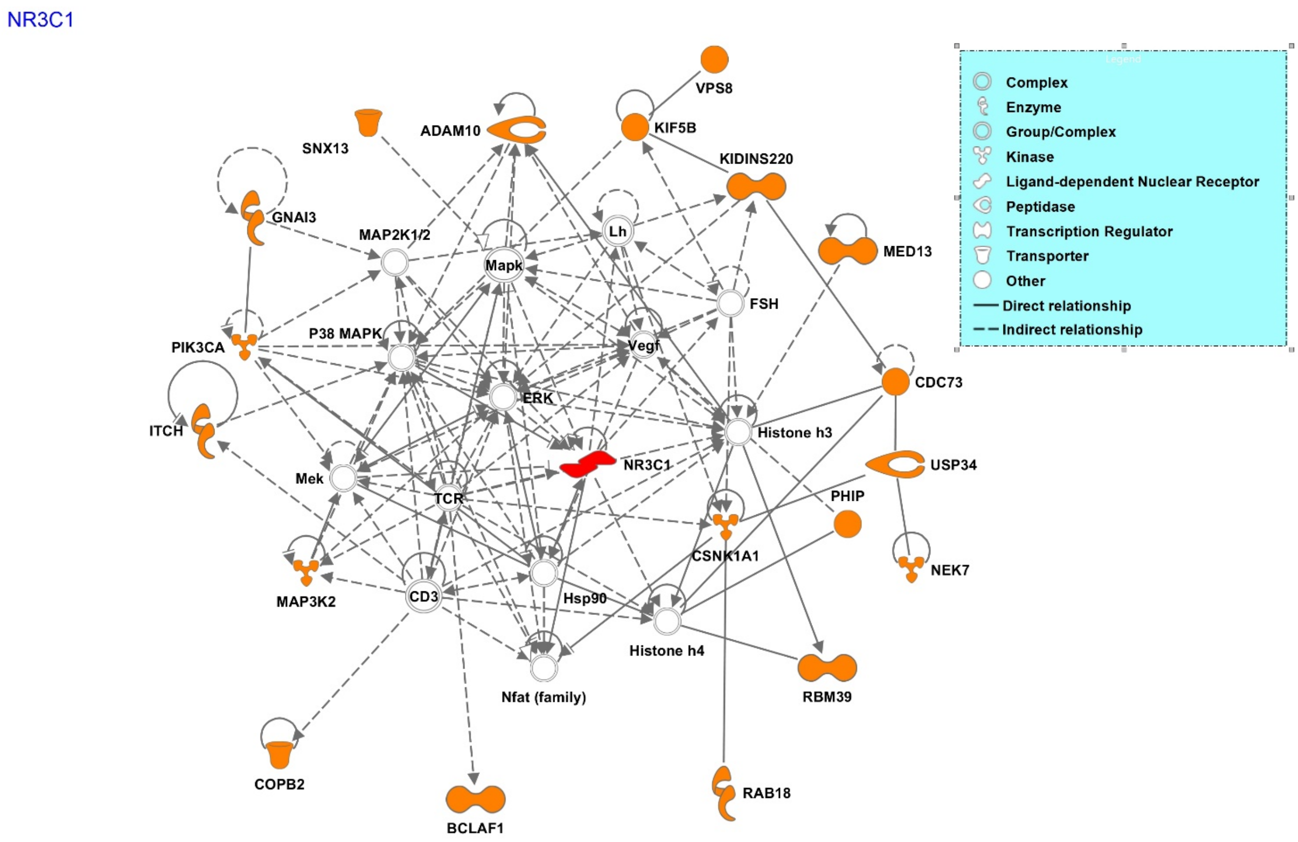

© 2000-2021 QIAGEN. All rights reserved.

Figure S3. Top network calculated by IPA when GR (NR3C1) and its 50 topmost associated genes were subjected to «core analysis» including direct and indirect relationships and default settings/filters. Orange symbols indicate genes among the 50 topmost GR associated.
